# Cadherin-dependent adhesion is required for muscle stem cell niche anchorage and maintenance

**DOI:** 10.1242/dev.202387

**Published:** 2024-04-04

**Authors:** Margaret Hung, Hsiao-Fan Lo, Aviva G. Beckmann, Deniz Demircioglu, Gargi Damle, Dan Hasson, Glenn L. Radice, Robert S. Krauss

**Affiliations:** ^1^ Department of Cell, Developmental, and Regenerative Biology, Icahn School of Medicine at Mount Sinai, New York, NY 10029, USA; ^2^ Black Family Stem Cell Institute, Icahn School of Medicine at Mount Sinai, New York, NY 10029, USA; ^3^ Graduate School of Biomedical Sciences, Icahn School of Medicine at Mount Sinai, New York, NY 10029, USA; ^4^ Pathos AI, 600 West Chicago Avenue, Suite 510, Chicago, IL 60654, USA; ^5^ Bioinformatics for Next Generation Sequencing Core, Icahn School of Medicine at Mount Sinai, New York, NY 10029, USA; ^6^ The Tisch Cancer Institute, Icahn School of Medicine at Mount Sinai, New York, NY 10029, USA; ^7^ Department of Oncological Sciences, Icahn School of Medicine at Mount Sinai, New York, NY 10029, USA; ^8^ Cardiovascular Research Center, Department of Medicine, Division of Cardiology, Alpert Medical School of Brown University, Providence, RI 02903, USA

**Keywords:** Muscle stem cell, Stem cell niche, Quiescence, Cell adhesion, Cell signaling

## Abstract

Adhesion between stem cells and their niche provides stable anchorage and signaling cues to sustain properties such as quiescence. Skeletal muscle stem cells (MuSCs) adhere to an adjacent myofiber via cadherin-catenin complexes. Previous studies on N- and M-cadherin in MuSCs revealed that although N-cadherin is required for quiescence, they are collectively dispensable for MuSC niche localization and regenerative activity. Although additional cadherins are expressed at low levels, these findings raise the possibility that cadherins are unnecessary for MuSC anchorage to the niche. To address this question, we conditionally removed from MuSCs β- and γ-catenin, and, separately, αE- and αT-catenin, factors that are essential for cadherin-dependent adhesion. Catenin-deficient MuSCs break quiescence similarly to N-/M-cadherin-deficient MuSCs, but exit the niche and are depleted. Combined *in vivo*, *ex vivo* and single cell RNA-sequencing approaches reveal that MuSC attrition occurs via precocious differentiation, re-entry to the niche and fusion to myofibers. These findings indicate that cadherin-catenin-dependent adhesion is required for anchorage of MuSCs to their niche and for preservation of the stem cell compartment. Furthermore, separable cadherin-regulated functions govern niche localization, quiescence and MuSC maintenance.

## Teaser

**Summary:** Genetic ablation of cadherin-based adhesion in skeletal muscle stem cells triggers activation, niche exit, precocious differentiation and subsequent depletion of the stem cell pool.

## INTRODUCTION

Adult stem cells reside in a specialized microenvironment, or niche, that supplies signals to sustain specific cellular properties (Fuchs and Blau, 2020). A general feature provided by the niche is anchorage of stem cells via cell-cell and/or cell-extracellular matrix adhesion (Chen et al., 2013; Parsons et al., 2010; Polisetti et al., 2016; Schüler et al., 2022). Niche-based adhesion in turn supports stable proximity of stem cells to biochemical and biomechanical signals, the nature of which may vary between tissues and stem cell types. Skeletal muscle stem cells (MuSCs, also called satellite cells) are localized between a myofiber and an enwrapping basal lamina (Hung et al., 2023). During homeostasis, MuSCs are quiescent, but in response to muscle injury they activate, proliferate, differentiate and fuse with each other or with existing myofibers to repair the damage (Hardy et al., 2016; Schmidt et al., 2019). They also self-renew and re-occupy the niche in regenerated muscle.

Niche localization induces polarity in MuSCs: laminin-binding integrins are found basally and cadherins are present apically, the latter being the cell surface where MuSCs are in direct contact with myofibers (Goel et al., 2017; Krauss et al., 2017; Rozo et al., 2016). α7β1-integrin is the major laminin receptor in MuSCs, and conditional genetic removal of β1-integrin from these cells results in loss of cell polarity and eventual MuSC attrition due to precocious differentiation (Rozo et al., 2016). Despite this perturbance, β1-integrin-deficient MuSCs remain under the basal lamina. Therefore, β1-integrin-mediated basal adhesion is required for maintenance of the MuSC compartment, but additional mechanisms are sufficient to retain MuSCs in the niche.

The roles of apical adhesion and cadherins in MuSCs are less clear. Cadherins are calcium-dependent homophilic cell-cell adhesion molecules that regulate tissue integrity and specific morphogenetic movements (Yap et al., 2018; Mège and Ishiyama, 2017). Classical cadherins have a cytoplasmic domain bound directly by β- and/or γ-catenin, which in turn bind to α-catenin. α-Catenin binds directly to F-actin or to adaptor proteins that bind F-actin, forming molecular complexes that provide stable cell-cell cohesion and transmit tensile forces from the exterior to the interior of the cell (Yap et al., 2018). MuSCs express multiple catenin-binding cadherins, including M-cadherin, N-cadherin and VE-cadherin, each localized to the MuSC apical membrane (Fukada et al., 2007; Goel et al., 2017). M-cadherin (encoded by *Cdh15*) expression is highly enriched in the skeletal muscle lineage, and *Cdh15* mRNA is expressed at much higher levels than *Cdh2* or *Cdh5* mRNAs (encoding N-cadherin and VE-cadherin, respectively) in quiescent MuSCs (Yue et al., 2020). Surprisingly, germline mutation of *Cdh15* in mice did not result in an obvious phenotype in skeletal muscle development, MuSC number or regeneration (Goel et al., 2017; Hollnagel et al., 2002). In contrast, conditional mutation of *Cdh2* in MuSCs rendered the cells prone to break quiescence and enter a state of partial activation, without compromising cell polarity or niche localization (Goel et al., 2017). Furthermore, MuSCs lacking *Cdh2* are proficient in regeneration and self-renewal (Goel et al., 2017). Combined loss of *Cdh15* and *Cdh2* exacerbated the phenotypes associated with loss of *Cdh2* alone, but double-mutant cells also remain in the niche and are functional in response to injury (Goel et al., 2017). Therefore, the most abundantly expressed cadherin, M-cadherin, is dispensable for MuSC function, whereas N-cadherin is expressed at much lower levels, yet it is required to prevent MuSCs from entering the activation process in the absence of injury. The role of N-cadherin in maintaining MuSC quiescence is linked to its function in promoting outgrowth and/or maintenance of cellular projections that apparently act as sensors of the niche (Kann et al., 2022).

VE-cadherin is present at the apical membrane of MuSCs lacking M- and N-cadherin (Goel et al., 2017). Additionally, quiescent MuSCs express RNA for at least one additional catenin-binding cadherin, albeit at low levels (R-cadherin, which is encoded by *Cdh4*) (Yue et al., 2020). Despite the presence of cadherins other than M- and N-cadherin, these findings raise the possibility that any cadherin-based adhesion may be dispensable for anchorage of MuSCs to their niche. Integrin-mediated basal adhesion and/or alternative mechanisms of apical adhesion may be sufficient for niche localization, even in the absence of the most abundant cadherin (M-cadherin) and a cadherin required for quiescence (N-cadherin). To address these possibilities, we assessed the consequences of depriving MuSCs of all ability to form cadherin-based adhesion structures. Simultaneous genetic removal of all expressed catenin-binding cadherins would require removing at least four cadherins, so we used conditional mutagenesis to target catenins, which are essential components of cadherin-based adhesion complexes. We separately removed two pairs of redundant catenins from MuSCs: β- and γ-catenin, and αE- and αT-catenin. We report here that MuSCs lacking these catenin pairs, and thereby deprived of cadherin-based adhesion, spontaneously activate and exit the niche, entering the interstitial space between myofibers. These cells are lost over time to what appears to be a single fate: precocious differentiation, re-entry to the niche and fusion to myofibers. Therefore, cadherin-based cell-cell adhesion is required for niche localization and preservation of MuSCs, and cadherins expressed at low levels appear to be sufficient for this stem cell property. Furthermore, the requirement for cadherins in MuSC niche localization and quiescence are distinguishable.

## RESULTS

### Conditional deletion of α-catenins or β- and γ-catenin results in MuSC attrition

**Fig. 1.**
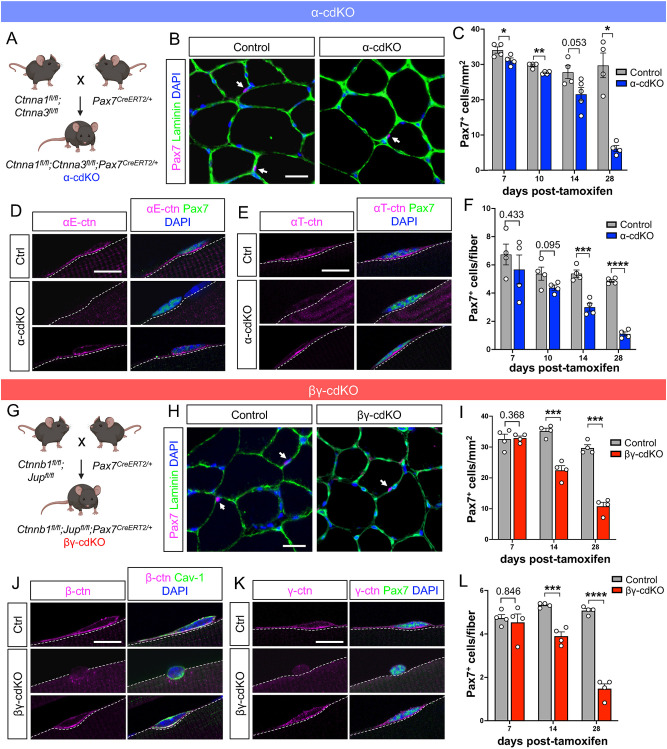
**Catenins are apically enriched in quiescent MuSCs and necessary for MuSC maintenance.** (A) Breeding scheme for α-cdKO mice: mice carrying homozygous floxed alleles for αE-catenin (*Ctnna1*) and αT-catenin (*Ctnna3*) loci were paired with mice carrying a MuSC-specific *Pax7*-driven CreERT2 (*Pax7^CreERT2^*) allele to yield α-cdKO mice. Created with BioRender. (B-F) TA muscle sections from control and α-cdKO mice at various time points were immunostained (Pax7, magenta; laminin, green; DAPI, blue) and Pax7-expressing MuSCs were labeled (B; white arrows) and quantified (C). Immunostaining of single myofibers (αE- or αT-catenin, magenta; caveolin 1 or Pax7, green; DAPI, blue) was used to assess the presence of αE- and αT-catenin protein (D,E) and MuSC attrition (F) in α-cdKO mice. Representative images of control and α-cdKO TA muscle sections and single myofibers were isolated from 28 DPT immunofluorescence analyses. (G) Breeding scheme for βγ-cdKO mice: mice carrying homozygous floxed alleles for β-catenin (*Ctnnb1*) and γ-catenin (*Jup*) loci were paired with mice carrying a MuSC-specific *Pax7*-driven CreERT2 allele (*Pax7^CreERT2^*) to yield βγ-cdKO mice. Created with BioRender. (H-L) TA muscle sections from control and βγ-cdKO mice at various time points were immunostained (Pax7, magenta; laminin, green; DAPI, blue) and Pax7-expressing MuSCs were labeled (H; white arrows) and quantified (I). Immunostaining of single myofibers (β- or γ-catenin, magenta; Pax7, green; DAPI, blue) was used to assess presence of β- and γ-catenin protein (J,K) and MuSC attrition (L) in βγ-cdKO mice. Representative images of control and βγ-cdKO TA muscle sections and single myofibers were isolated from 28 DPT immunofluorescence analyses. Scale bars: 25 μm in B,H; 10 μm in D,E,J,K. Each data point represents the average from ten fields (TA muscle sections) or at least ten myofibers (EDL single myofibers) from each animal. *n*≥4 animals per genotype per timepoint. Data are mean±s.e.m. with comparisons made using a two-tailed unpaired Student's *t*-test. *P<0.05, **P<0.01, ***P<0.001, ****P<0.0001.

Cadherins require β- or γ-catenin plus α-catenin to form stable cell adhesion junctions (Mège and Ishiyama, 2017). To determine the effects of loss of cadherin-based adhesion complexes, conditional mutagenesis approaches were taken. MuSCs express two isoforms of α-catenin, αE and αT, which are respectively encoded by *Ctnna1* and *Ctnna3* (Yue et al., 2020). Mice homozygous for floxed alleles for both genes were bred with mice that carried a MuSC-specific *Pax7^CreERT2^* allele, yielding *Ctnna1^fl/fl^;Ctnna3^fl/fl^;Pax7^CreERT2^* mice (referred to as α-cdKO mice; Fig. 1A). MuSCs express both β- and γ-catenin, encoded by *Ctnnb1* and *Jup*, respectively (Goel et al., 2017; Yue et al., 2020). A similar approach was taken to produce *Ctnnb1^fl/fl^;Jup^fl/fl^;Pax7^CreERT2^* mice (referred to as βγ-cdKO mice; Fig. 1G). Two- to 3-month-old mice were treated with tamoxifen (TMX) for 5 days to activate Cre and studied at various time points afterwards for catenin protein expression and MuSC homeostasis, as assessed by number of Pax7^+^ cells.

α-cdKO mice displayed a steady decline in the number of Pax7^+^ MuSCs, beginning ∼10 days after the last TMX injection. This was observed in cross-sections of tibialis anterior (TA) muscle over a 28-day time course, at which point α-cdKO mice had lost 78% of Pax7^+^ MuSCs when compared with their control counterparts (Fig. 1B,C). Very similar results were obtained when quantifying Pax7^+^ MuSCs on single myofibers prepared from extensor digitorum longus (EDL) muscles (Fig. 1D-F). Single myofiber preparations were used to assess expression of αE- and αT-catenin by immunofluorescence. Across all time points, control MuSCs were uniformly positive for αE- and αT-catenin proteins, which were enriched at the apical membrane of each cell (Fig. 1D,E, Fig. S1A). At each time point post-TMX, the majority of remaining MuSCs were positive for one or the other α-catenin protein, indicating that MuSCs that lose both α-catenin proteins did not accumulate (Fig. S1A). As MuSC attrition was negligible at 7 days post-TMX (DPT), a time when recombination is presumably largely or fully complete, we infer that α-catenin protein perdurance must occur to a significant extent, and that as catenin levels drop below a certain threshold via protein turnover, cells are lost. Of the 22% of MuSCs remaining on EDL myofibers at 28 DPT, 82% were αE-catenin immunoreactive and 78% were αT-catenin immunoreactive, whereas the rest were negative (Fig. 1D,E). The fraction of MuSCs that remained α-catenin^+^ at this time point very likely failed to recombine all four floxed alleles, or some sufficient number of alleles, to cause cell loss. α-cdKO muscles were grossly normal, with no change in overall myofiber size compared with controls (Fig. S1B). This suggested that a steady decline of α-cdKO MuSCs over time could be due to loss of apical cadherin-based adhesion.

We performed parallel analyses with βγ-cdKO mice. The phenotype of βγ-cdKO mice at 28 DPT was similar to that of α-cdKO mice, with loss of 64% and 71% of Pax7^+^ MuSCs in cross-section analyses of TA muscle and on single EDL myofibers, respectively (Fig. 1H,I,L). Likewise, most MuSCs remained positive for either β- or γ-catenin at each time point analyzed (Fig. 1J,K, Fig. S1C). It again seems likely that the fraction of MuSCs remaining β- and/or γ-catenin^+^ at 28 DPT had failed to recombine all four floxed alleles, leaving enough residual protein to prevent MuSC loss. βγ-cdKO muscles also remained grossly normal, with no changes in average myofiber size at 28 DPT (Fig. S1D).

β-Catenin plays crucial roles in both cadherin-based adhesion and canonical Wnt signaling (van der Wal and van Amerongen, 2020). γ-Catenin has overlapping function with β-catenin in binding classical cadherins but is not involved in Wnt signaling; it is, however, crucial for formation of desmosomes, another type of cell-cell junction comprising desmosomal cadherins (Green et al., 2019; Kowalczyk and Green, 2013). β-Catenin has been conditionally removed from adult MuSCs in previous reports, and it did not lead to MuSC attrition at homeostasis (Murphy et al., 2014; Rudolf et al., 2016). Additionally, quiescent MuSCs did not display canonical Wnt signaling activity. These results suggest that the phenotype of βγ-cdKO mice is due to loss of cell adhesion complexes, not due to perturbance of Wnt signaling. To provide additional evidence for this contention, we studied mice lacking only γ-catenin in MuSCs (γ-cKO mice). In contrast to βγ-cdKO mice, γ-cKO mice did not display any loss of Pax7^+^ MuSCs at 28 DPT, despite 94% of MuSCs on single myofibers lacking γ-catenin protein expression (Fig. S2). These results indicate that removal of both β- and γ-catenins is required for MuSC attrition. Combined with the similar phenotype, over a similar time course, seen in α-cdKO mice, our findings strongly suggest that loss of cadherin-based cell-cell junctions, rather than other functions provided by the various catenins, is the likely major cause of MuSC attrition in these mice.

### α-cdKO and βγ-cdKO mice display incomplete muscle regeneration and do not efficiently reoccupy the niche

**Fig. 2.**
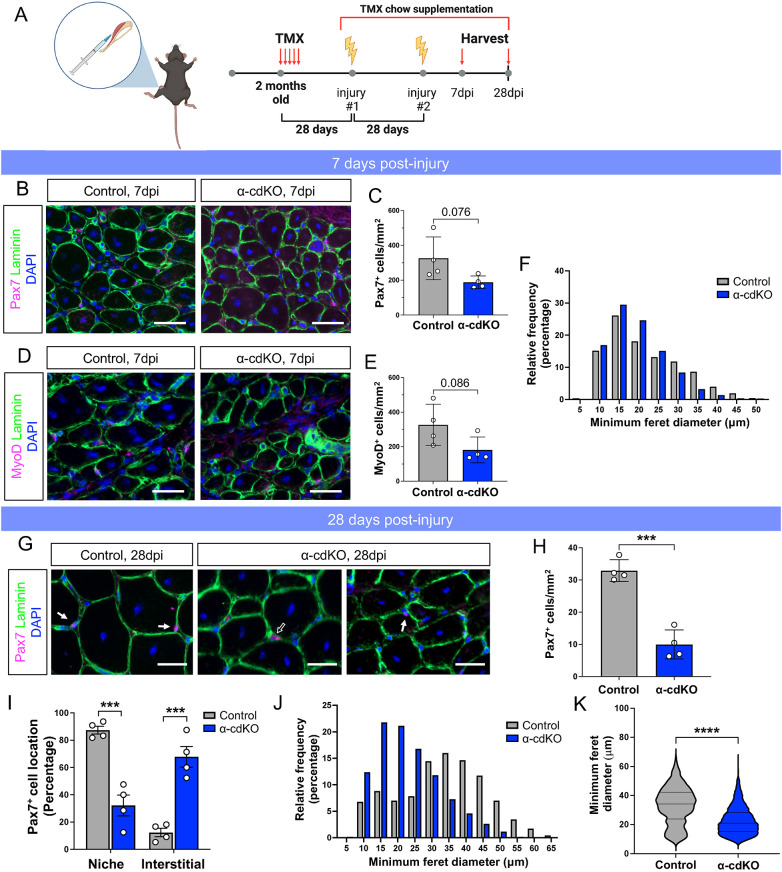
**α-cdKO mice have modest defects in muscle regeneration.** (A) Double BaCl_2_ injury model. α-cdKO mice were injected with tamoxifen for 5 consecutive days to recombine α-catenin alleles. At 28 DPT, mice were placed on tamoxifen-supplemented chow, and the first injury was applied to the hindlimb by an intramuscular injection of 1.2% BaCl_2_ in saline. After 28 days of recovery, a second injury was applied. Muscle was harvested and snap frozen at 7 and 28 dpi for analysis. Created with BioRender. (B-F) At 7 dpi, TA muscle sections from control and α-cdKO mice were immunostained (Pax7 or MyoD, magenta; laminin, green; DAPI, blue) for Pax7^+^ (B,C) and MyoD^+^ (D,E) cells to assess MuSC and myogenic progenitor numbers during early regeneration. (F) Myofiber size was quantified by minimum feret diameter. (G-K) At 28 dpi, TA muscle sections from control and α-cdKO mice were immunostained (G; Pax7, magenta; laminin, green; DAPI, blue) to assess MuSC numbers (H) and location (I) after complete regeneration. (J,K) Myofiber size was quantified by minimum feret diameter (J) with a statistical analysis in median myofiber size carried out using a two-tailed Mann–Whitney test (K). Scale bars: 25 μm in B,D,G. White arrows indicate a cell under the basal lamina; black arrow indicates an interstitial cell. Each data point represents the average from ten fields from each animal. *n*=4 animals per genotype. Data are mean±s.e.m. with comparisons made using a two-tailed unpaired Student's *t*-test, unless otherwise noted. ***P<0.001, ****P<0.0001.

MuSCs are essential for muscle regeneration (Lepper et al., 2011; Murphy et al., 2011; Sambasivan et al., 2011), but mice harboring only 10-20% of the total MuSC pool are still capable of muscle injury repair, indicating that regeneration is not dependent on large numbers of MuSCs (von Maltzahn et al., 2013). To assess whether the reduced MuSC populations in α-cdKO and βγ-cdKO mice were capable of regenerating muscle, we performed two consecutive BaCl_2_ injuries (Fig. 2A). Mice were maintained on TMX chow to continue Cre recombination stimulus during the regeneration period, and regeneration was assessed in both lines at 7 and 28 days post-injury (dpi) to observe early and complete regeneration, respectively.

In α-cdKO mice, the numbers of both Pax7^+^ MuSCs and MyoD^+^ myogenic precursor cells trended downward at 7 dpi when compared with control mice, but fell short of *P*<0.05 (Fig. 2B-E), which was closely mirrored in βγ-cdKO mice (Fig. S3A-D). Similarly, there was a slight shift toward smaller myofiber minimum feret diameter measurements in mutants (Fig. 2F, Fig. S3E). At 28 dpi, the number of Pax7^+^ MuSCs in α-cdKO and βγ-cdKO mice had returned to a similar density to that at 28 DPT in uninjured mice (Fig. 1C,I), a ∼70% decrease relative to control mice (Fig. 2G,H, Fig. S3F,G). These data suggest that the initial diminished pool of Pax7^+^ MuSCs in α-cdKO and βγ-cdKO mice had self-renewed but did not expand to refill the normal complement of stem cells. In contrast to what was observed at 7 dpi, myofiber minimum feret diameter was strongly shifted towards smaller sizes at 28 dpi (Fig. 2J,K, Fig. S3H), indicating a suboptimal regeneration response. Furthermore, 68% of α-cdKO MuSCs were localized in the interstitial space at 28 dpi, compared with 12% in control MuSCs (Fig. 2G,I). This phenomenon was also observed in βγ-cdKO mice; 44% of βγ-cdKO MuSCs were found in the interstitial space, compared with only 13% of control MuSCs (Fig. S3F,I). Wild-type levels of catenin expression are therefore required for MuSCs to reoccupy the niche after injury. Regenerated muscles in α-cdKO and βγ-cdKO mice also displayed an increase in collagen deposition, as detected by Picrosirius Red staining (Fig. S3J,K). This may be due to the presence of an increased number of smaller myofibers, thereby increasing circumferential fiber surface area per field, rather than a primary regenerative defect (Figs S3H,J,K and S4). Taken together, these data demonstrate that, whereas α-cdKO and βγ-cdKO mice maintained a number of Pax7^+^ MuSCs normally capable of mounting a full regenerative response (von Maltzahn et al., 2013), the remaining α-cdKO and βγ-cdKO MuSCs provided only a diminished regenerative response. This phenotype may arise from a combination of MuSC depletion and reduced function of the remaining partially catenin-deficient MuSCs.

### α-cdKO MuSCs do not adopt non-myogenic fates

Initial homeostatic characterization and injury studies in α-cdKO and βγ-cdKO mice resulted in strikingly similar phenotypes, implicating loss of cadherin-catenin-based adhesion as an initiating event of MuSC attrition. To identify the fate of MuSCs lacking catenins during homeostasis, we focused on α-cdKO mice, as a higher percentage of cells were affected after TMX treatment than in βγ-cdKO mice. We performed single cell RNA-sequencing on purified α-cdKO MuSCs at 14 DPT, a timepoint midway into the process of MuSC attrition. A tdTomato reporter allele was crossed onto the α-cdKO line, and hindlimb muscles from 3-month-old control and α-cdKO mice were dissociated into single cell suspensions for FACS isolation of tdTomato^+^ cells. This protocol avoided sorting MuSCs based on expression of cell surface markers, which could have changed upon loss of α-catenins. Importantly, any cells that lost myogenic identity would still be captured for analysis.

**Fig. 3.**
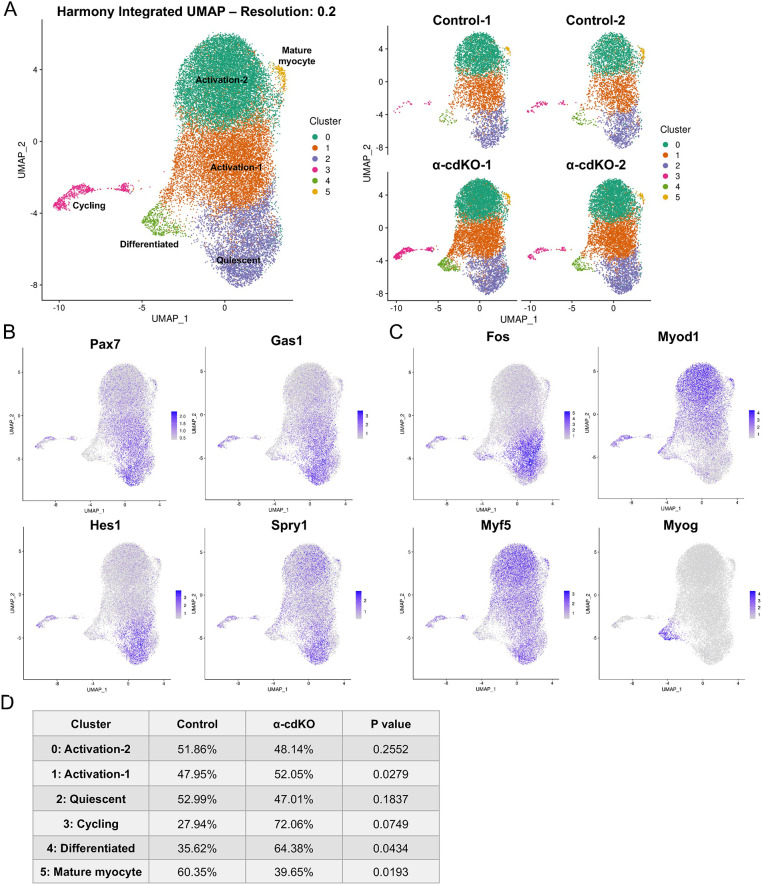
**α-cdKO MuSCs do not adopt non-myogenic lineage fates.** (A) Single cell RNA-sequencing analysis of tdTomato^+^ cells from control and α-cdKO mice at 14 DPT revealed six distinct cell clusters after Harmony integration, with no cluster being unique to either genotype. (B,C) Gene expression of quiescence-associated (B) and activation- or differentiation-associated (C) factors illustrated a continuum of the quiescence-to-activation transition present in both genotypes. (D) Clusters were categorized based on top differentially expressed genes; genotype contribution to each cluster was normalized to the total number of cells analyzed.

Overall quality metrics assessed via CellRanger confirmed sample robustness before Harmony integration using all samples followed by unsupervised clustering of cells and non-linear dimensionality reduction via uniform manifold approximation and projection (UMAP) (Fig. S5A,B) (Korsunsky et al., 2019; McInnes et al., 2018). Eight unsupervised clusters were identified, none of which was unique to control or α-cdKO MuSCs (Fig. S5B). After integration, cells were filtered for MuSC and myogenic progeny identity using UniCell Deconvolve unsupervised cell type annotations, with the great majority of cells identified as skeletal muscle stem cells or myogenic precursors (94%; 27,128 out of 28,790 total cells). Remaining cells were classified by UniCell as ‘striated muscle’ and excluded from downstream analyses (Fig. S5C) (Charytonowicz et al., 2023). Unsupervised clustering of MuSCs identified six clusters identified in both control and α-cdKO samples, showing similar characteristics to previously reported datasets (Fig. 3, Fig. S5D,E). A cluster of cells expressed transcripts for *Pax7*, *Gas1*, the Notch target *Hes1* and receptor tyrosine kinase inhibitor *Spry1*, which revealed the isolation of a population similar to quiescent MuSCs *in vivo* (cluster 2, Fig. 3A,B) (Dell'Orso et al., 2019; Machado et al., 2017). Many cells expressed the immediate-early gene *Fos* within the same cluster, indicative of MuSCs in the earliest state of activation, likely due to the isolation process (Fig. 3A,C) (Almada et al., 2021; Kann et al., 2022; Machado et al., 2017; Van Velthoven et al., 2017). It is well established that the process of isolating and sorting MuSCs induces a stress response in, and activation of, MuSCs (Machado et al., 2017, 2021; Van Velthoven et al., 2017). We also observed this phenomenon (Fig. S5D). *Myod1* and *Myf5* expression was found in multiple clusters, with varying degrees of expression and overlapped with *Pax7* expression and progressive MuSC activation (clusters 0 and 1; Fig. 3A,C). In contrast, *Myog* expression, found only in cells committed to differentiate, was found in a single cluster (cluster 4); these cells comprise a greater fraction in α-cdKO cells than in control cells (*P*=0.0434) (Fig. 3D). Overall, the changes in gene expression associated with MuSC isolation made it difficult to identify changes in early activation that occurred in α-cdKO MuSCs. However, this analysis of tdTomato-marked cells demonstrated that α-cdKO MuSCs did not adopt non-myogenic cell fates, indicating that they were lost by an alternative mechanism.

### α-cdKO MuSCs undergo activation in the absence of injury

**Fig. 4.**
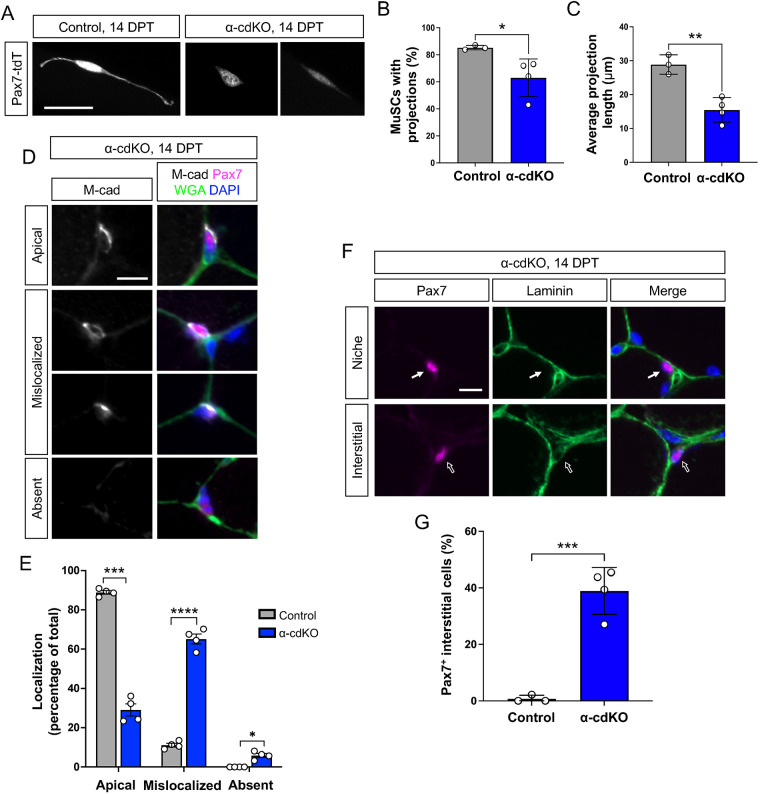
**Loss of α-catenins compromises cadherin-based adhesion and leads to escape from the niche.** (A-C) Endogenous tdTomato reporter fluorescence from MuSCs on isolated single EDL myofibers from control and α-cdKO mice at 14 DPT (A) was used to quantify the proportion of MuSCs maintaining cytoplasmic projections (B) and the average length of remaining projections (C). (D,E) At 14 DPT, TA muscle sections from control and α-cdKO mice at 14 DPT were immunostained (M-cadherin, grey; Pax7, magenta; WGA, green; DAPI, blue) to assess M-cadherin localization in Pax7^+^ MuSCs (D,E). (F,G) At 14 DPT, TA muscle sections from control and α-cdKO mice at 14 DPT were immunostained (Pax7, magenta; laminin, green; DAPI, blue) (F; white arrows indicate Pax7^+^ cells) to assess MuSC localization (G). Scale bars: 25 μm in A,F; 10 μm in D. Each data point represents the average quantified from *n*≥30 cells from each animal (TA muscle sections) or at least ten myofibers (EDL single myofibers) from each animal. *n*=4 animals per genotype. Data are mean±s.e.m. with comparisons made using two-tailed unpaired Student's *t*-test. *P<0.05, **P<0.01, ***P<0.001, ****P<0.0001.

Quiescent MuSCs have long heterogeneous projections and retraction of these structures is a very early response to muscle injury (Kann et al., 2022; Ma et al., 2022; Verma et al., 2018). Consistent with this observation, MuSCs characterized as being in a state of partial activation (e.g. *Cdh2*-mutant MuSCs and MuSCs in G_alert_) either lack or have shorter projections (Kann et al., 2022; Goel et al., 2017; Rodgers et al., 2014). To visualize and quantify MuSC projections, we used α-cdKO mice carrying a tdTomato reporter, and isolated single myofibers from these and control mice carrying the reporter at 14 DPT. α-cdKO MuSCs had fewer and shorter cellular projections than control MuSCs, consistent with the possibility of their having initiated the activation process in the absence of injury (Fig. 4A-C).

We next assessed cadherin protein distribution and niche localization. TA muscle sections were immunostained for Pax7 and M-cadherin, the latter serving as a readout for polarized distribution of cadherin-based junctions in MuSCs (Fig. 4D,E). In 89% of control MuSCs, M-cadherin was enriched at the apical membrane, the cell surface in direct contact with a myofiber. In contrast, 65% of α-cdKO MuSCs had M-cadherin present throughout the plasma membrane or enriched on the basal side of the cell. Additionally, 6% of mutant cells completely lacked M-cadherin signal. Therefore, in the absence of α-catenins, MuSCs were unable to maintain polarized apical localization of the major cadherin protein expressed in these cells. Consistent with loss of this characteristic property of MuSCs, 39% of Pax7^+^ α-cdKO MuSCs had exited the niche and were detected in the interstitial space between myofibers, whereas virtually all control MuSCs were found within the niche, under the basal lamina (Fig. 4F,G).

**Fig. 5.**
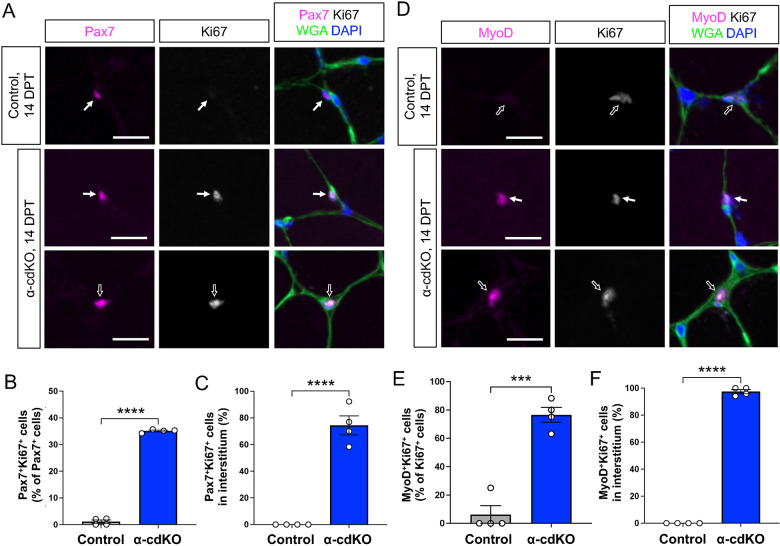
**α-cdKO MuSCs activate and enter the cell cycle in the absence of injury.** (A-C) TA muscle sections from control and α-cdKO mice at 14 DPT were immunostained (Pax7, magenta; Ki67, grey; WGA, green; DAPI, blue) (A) to assess the incidence of Pax7^+^Ki67^+^ cells (B) and their location (C). (D-F) TA muscle sections from control and α-cdKO mice at 14 DPT were immunostained (MyoD, magenta; Ki67, grey; WGA, green; DAPI, blue) (D) to assess the incidence of MyoD^+^Ki67^+^ cells among all Ki67^+^ cells (E) and their location (F). Scale bars: 10 μm in A,D. Each data point represents the average quantified from *n*≥30 cells from each animal. White arrows indicate a cell under the basal lamina; black arrows indicate an interstitial cell. *n*=4 animals per genotype. Data are mean±s.e.m. with comparisons made using a two-tailed unpaired Student's *t*-test. ***P<0.001, ****P<0.0001.

To further assess whether α-cdKO MuSCs became activated and entered the cell cycle, TA sections were stained for Pax7 and for the MuSC cell cycle and activation marker Ki67. Although Pax7^+^ MuSCs in control mice rarely expressed Ki67, 35% of α-cdKO MuSCs were Ki67^+^ (Fig. 5A,B). Furthermore, 74% of Pax7^+^Ki67^+^ MuSCs were present in the interstitial space, outside the basal lamina (Fig. 5A,C). Approximately 77% of Ki67^+^ α-cdKO MuSCs were also positive for the activation and myogenic progenitor cell marker MyoD, with 97% of MyoD^+^Ki67^+^ cells present in the interstitial space (Fig. 5D-F). Taken together, α-cdKO MuSCs exhibited a range of phenotypes at 14 DPT, consistent with sequencing data (Fig. 3). Although nearly all MyoD^+^Ki67^+^ cells had exited the niche, a fraction of α-cdKO Pax7^+^ MuSCs remained under the basal lamina and did not express Ki67 (Fig. 5A,B). Some of these latter cells may not have undergone complete loss of α-catenins, either due to incomplete Cre-mediated recombination or to perdurance of a sufficient level of α-catenin protein to maintain cadherin-dependent adhesion. Alternatively, distinct, but not fully sufficient, adhesion mechanisms may exist that lead to asynchronous MuSC activation and niche exit in the absence of α-catenins.

### α-cdKO MuSCs progress through the myogenic lineage and are lost to precocious differentiation and fusion

A large fraction of α-cdKO MuSCs at 14 DPT displayed features of activated MuSCs. Attrition of these cells had begun by this point and continued for another 14 days (Fig. 1). We examined possible mechanisms by which α-cdKO MuSCs were lost, including apoptosis, senescence, and differentiation and fusion to myofibers. To assess apoptosis, TA muscle sections were stained for cleaved caspase 3 and analyzed by TUNEL assay, with sections of intestinal epithelium from the same animals as a positive control for the former and DNase I-treated TA sections as a positive control for the latter. In control and α-cdKO muscles, cell death was not detected by either method (Fig. S6A,B). To address the possibility of α-cdKO MuSCs entering senescence, senescence-associated β-galactosidase expression in control and α-cdKO mice was analyzed; no β-galactosidase expression was detected in either line, in contrast to a positive control at 7 dpi (Moiseeva et al., 2023) (Fig. S6C). Therefore, α-cdKO MuSCs were not lost due to apoptosis or senescence.

**Fig. 6.**
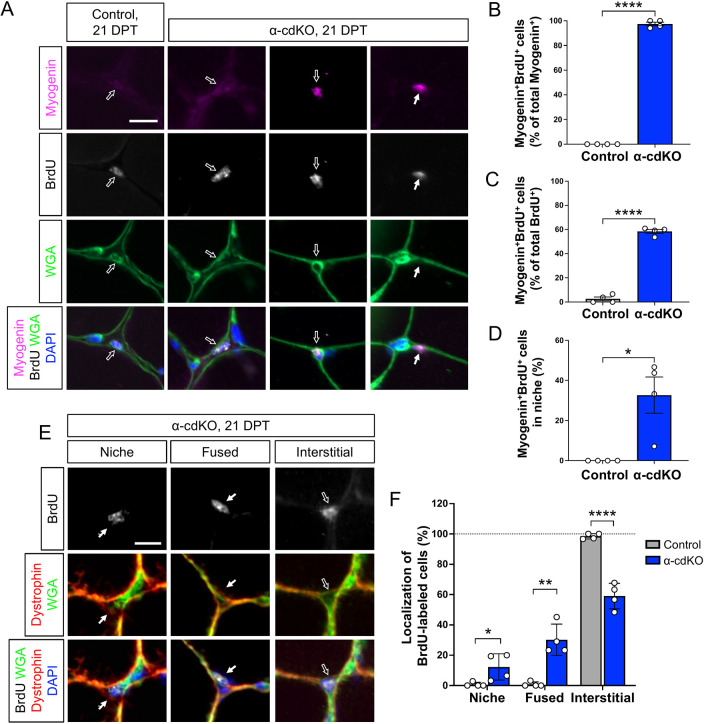
**α-cdKO MuSCs are lost to precocious differentiation and fusion.** (A-D) TA muscle sections from control and α-cdKO mice at 21 DPT were immunostained (A; myogenin, magenta; BrdU, grey; WGA, green; DAPI, blue) to study cell cycle progression into S phase by BrdU incorporation and to examine the precocious differentiation of myogenic cells by expression of myogenin (B-D). (B-D) Cycling myogenic cells (B,C) and location of myogenin^+^BrdU^+^ cells (D) were quantified. (E,F) TA muscle sections from control and α-cdKO mice at 21 DPT were immunostained for sarcolemma marker dystrophin (red) and basal lamina marker WGA (green) (E) to assess whether actively cycling myogenic cells under the basal lamina were able to initiate fusion into the myofiber (F). Scale bars: 10 μm in A,E. Each data point represents quantification of *n*≥30 cells (TA muscle sections) from each animal. White arrows indicate a cell under the basal lamina; black arrows indicate an interstitial cell. *n*=4 animals per genotype. Data are mean±s.e.m. with comparisons made using a two-tailed unpaired Student's *t*-test. *P<0.05, **P<0.01, ****P<0.0001.

To validate the increased expression of *Myog* seen in our sequencing data and test for loss of α-cdKO MuSCs via differentiation and fusion with myofibers, we marked MuSC nuclei with a BrdU pulse-chase strategy. α-cdKO mice received BrdU injections three times a day for 5 consecutive days (10-14 DPT) and TA muscles were harvested at 21 DPT then immunostained for BrdU and markers of the muscle lineage (Fig. S7A). In control mice, 4% of Pax7^+^ cells incorporated BrdU, whereas 8% of Pax7^+^ cells in α-cdKO muscle did so, although this comparison fell short of *P*<0.05 (Fig. S7B,C). It is likely that at 21 DPT, few cells that incorporated BrdU would have remained Pax7^+^. Sections were therefore assessed for expression of myogenin, which drives terminal differentiation of muscle progenitor cells (Hernández-Hernández et al., 2017). Myogenin^+^ cells were rarely observed in TA muscle sections of control mice. In contrast, myogenin^+^ cells were abundant in α-cdKO mice and 97% of these cells were also BrdU^+^ (Fig. 6A,B). Most (58%) BrdU^+^ cells were also positive for myogenin (Fig. 6C). Interestingly, although almost all MyoD^+^Ki67^+^ cells in α-cdKO mice were located in the interstitial space, 33% of myogenin^+^BrdU^+^ cells were found under the basal lamina (Figs 5D,F and 6A,D). MyoD expression precedes and induces myogenin expression, suggesting that these latter cells have returned to the MuSC niche.

To determine whether α-cdKO MuSCs may fuse with their associated myofibers, we stained sections from 21 DPT mice for BrdU incorporation, dystrophin to mark the interior of the myofiber sarcolemma and wheat germ agglutinin (WGA) to label the basal lamina. In control mice, BrdU^+^ cells were infrequent and virtually all were located in the interstitial space [these cells are likely to be muscle-resident cells that divide at a measurable rate during homeostasis (e.g. fibroadipogenic progenitors or endothelial cells)] (Joe et al., 2010; Murphy et al., 2011; Verma et al., 2018; Wosczyna et al., 2019). In contrast, 59% of BrdU^+^ cells in α-cdKO muscle were located in the interstitial space, and as discussed above most of these expressed the muscle-specific marker myogenin. 12% of BrdU^+^ cells were found within the typical MuSC niche, sandwiched between the basal lamina and myofiber surface, whereas 30% were positive nuclei found under both WGA and dystrophin, having fused with the myofiber (Fig. 6E,F). We found no evidence to suggest that cells had diluted their incorporated BrdU via proliferation. BrdU dilution to below immunofluorescent detection limits has been quantified as requiring between two and five divisions (Cutler et al., 2022; Parretta et al., 2008; Wilson et al., 2008), yet no clusters of mononucleated BrdU^+^ cells were observed (Fig. 6A,E and Fig. S7) and, as described above, no changes to myofiber size were seen (Fig. S1B,D). These findings are therefore consistent with the hypothesis that α-cdKO MuSCs initiate differentiation in the interstitial space, then return to the niche and fuse with the myofiber. Furthermore, this appears to be the sole mechanism of MuSC attrition in α-cdKO mice.

## DISCUSSION

A variety of adult stem cells, including MuSCs, exist in a state of quiescence for extended periods of time (Fuchs and Blau, 2020). Quiescence is an actively maintained property, requiring multiple signals provided by the stem cell niche (Urbán and Cheung, 2021; van Velthoven and Rando, 2019). Cell-cell and cell-matrix adhesion between stem cells and their niche are important regulators of this process, promoting stable niche localization as well as specific signaling information (Hung et al., 2023; Schüler et al., 2022; Polisetti et al., 2016; Chen et al., 2013; Parsons et al., 2010). Maintenance of MuSC quiescence is important for stem cell function; when it is broken non-physiologically (e.g. in geriatric or genetically modified mice), it usually leads to loss of a functional MuSC pool and impaired regeneration (Evano and Tajbakhsh, 2018; Hong et al., 2022; Kimmel et al., 2020; Relaix et al., 2021). We have previously reported that genetic removal from MuSCs of M-cadherin, the major niche cadherin by expression level, had no effect on MuSC homeostasis. In contrast, removal of N-cadherin resulted in a propensity of MuSCs to asynchronously enter a state of partial activation, followed by progression through full activation, cell division, cell differentiation and fusion with myofibers (Goel et al., 2017). Combined loss of N- and M-cadherin exacerbated this phenotype (Goel et al., 2017). Despite a failure to maintain quiescence, MuSCs lacking both cadherins remained polarized and in a normal niche location, and they preserved regenerative and self-renewal capabilities. We attributed these unusual latter properties to yet additional cadherins expressed at lower levels than N- and M-cadherin (Yue et al., 2020). Nevertheless, these observations raised the possibility that although cadherins are clearly important for maintenance of MuSC quiescence, they may be dispensable for stable niche localization. Basal adhesion alone may be sufficient, and alternative apical adhesion mechanisms may also exist, including Notch ligand/receptor interactions and yet-to-be-explored factors such as protocadherins (Murata et al., 2014; Pancho et al., 2020). We therefore addressed the effects in MuSCs of complete loss of cadherin-based adhesion by genetically removing pairs of redundant catenin proteins that are required for cadherin function. Our findings demonstrate that cadherin-based adhesion is essential for stable niche localization of MuSCs, and that MuSCs are depleted in its absence. Furthermore, N-cadherin plays a specific role in preventing MuSCs from breaking quiescence, a role that is distinct from the requirement for cadherins in maintaining niche adhesion.

MuSC-specific removal of β- and γ-catenins or, separately, αE- and αT-catenins led to MuSC attrition over the course of 4 weeks in both lines. A fraction of MuSCs persisted at this and later timepoints but the majority of such cells were still positive for at least one of the target proteins, indicating incomplete Cre-mediated recombination or exceptionally long perdurance of the protein in a subset of MuSCs. Although these catenin pairs are essential for cadherin-dependent cell-cell adhesion, they also play additional roles in cell regulation. β-Catenin is a crucial signal transducer of canonical Wnt signaling, and γ-catenin plays a central role in organization of desmosomes via binding to desmosomal cadherins (Green et al., 2019; van der Wal and van Amerongen, 2020). However, canonical Wnt signaling is silent during MuSC quiescence, and MuSCs do not express desmosomal cadherins (Murphy et al., 2014; Parisi et al., 2015; Rudolf et al., 2016; Yue et al., 2020). Additionally, single genetic removal of β- or γ-catenin was without effect on MuSC numbers at homeostasis, whereas combined removal resulted in MuSC attrition (Murphy et al., 2014; Rudolf et al., 2016; this study). These results indicate that β- and γ-catenin share a function required for MuSC maintenance, and that this function is almost certainly classical cadherin-based adhesion. This conclusion is consistent with results from ablation of these two cadherin-binding proteins in heart muscle and in motor neuron pools during spinal cord development (Demireva et al., 2011; Swope et al., 2012). α-catenins also play roles in regulating signaling, but these are usually performed as components of adhesion complexes (Priya and Yap, 2015; Yap et al., 2018). For example, in some cell types, α-catenin binds YAP to prevent its dephosphorylation and nuclear translocation (Schlegelmilch et al., 2011; Silvis et al., 2011). However, YAP expression is induced later during MuSC activation, and expression of constitutively active YAP in adult MuSCs *in vivo* is insufficient to break quiescence (Judson et al., 2012; Tremblay et al., 2014). Release of YAP as a consequence of adhesion complex deficiency is therefore unlikely to explain the phenotype of α-cdKO mice. We therefore conclude that the primary defect in these mouse lines is a loss of cadherin-based adhesion in MuSCs and not other functions of catenins.

**Fig. 7.**
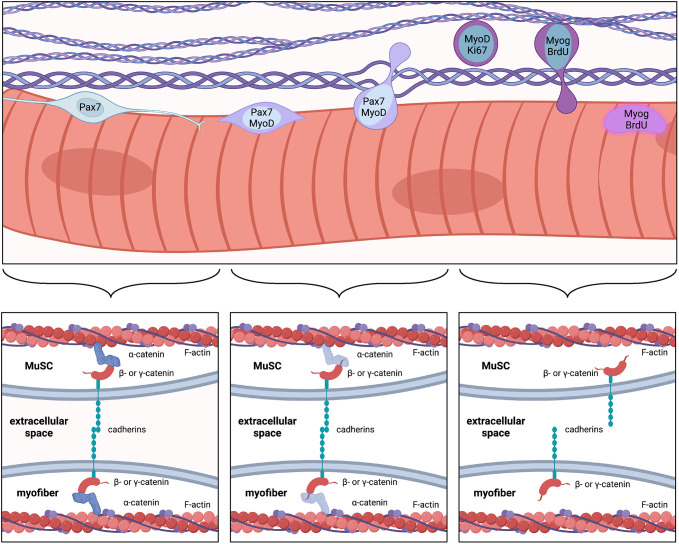
**Schematic representation of α-cdKO MuSC behavior.** Attrition of Pax7^+^ MuSCs in α-cdKO mice is due to the loss of apical cadherin-catenin-based adhesion to the myofiber. This results in cells that become activated in the absence of injury, exit the niche, enter the cell cycle and terminally differentiate to fuse into the myofiber. Created with BioRender.

It is intriguing that MuSCs lacking N- and M-cadherin, and α-cdKO MuSCs both appear to go through similar stages of activation and progression in the absence of injury, including expression of MyoD, entry to the cell cycle, differentiation and fusion with myofibers. However, N-/M-cadherin-deficient MuSCs remain in the niche and are not depleted, whereas α-cdKO MuSCs exit the niche and are depleted (Fig. 7). It is possible that the ability of N-/M-cadherin mutant MuSCs to stay under the basal lamina, due to a diminished but functional complement of classical cadherins, allows them to enter and slowly complete a normal activation process. In contrast, full loss of cadherin-based adhesion in α-cdKO MuSCs results in escape from the niche, where the interstitial microenvironment may lack signals required for normal activation and myogenic progression, ultimately leading to precocious differentiation and MuSC attrition. However, cadherin-dependent niche localization is not required for MuSC survival or maintenance of myogenic identity, as the sole fate of α-cdKO MuSCs appears to be differentiation and fusion with myofibers. The latter event requires these cells to recross the basal lamina. It is possible, in fact, that α-cdKO MuSCs cross the basal lamina multiple times before fusion. This may be related to expression of matrix metalloproteinases by activated MuSCs (Pallafacchina et al., 2010), but other mutants display similar unscheduled activation without exiting the niche (Bjornson et al., 2012; Mourikis et al., 2012; Rozo et al., 2016).

These observations raise fundamental questions: what are the adhesion requirements for MuSCs to adopt stable niche localization, and are they the same as those necessary to maintain quiescence? The basal lamina is mainly composed of laminin and collagen IV (Baghdadi et al., 2018; Schüler et al., 2022). β1-Integrin heterodimerizes with α7-integrin to form the major MuSC laminin receptor and is also a component of collagen receptors (Rozo et al., 2016; Schüler et al., 2022; Mashinchian et al., 2018). MuSC-specific mutation of β1-integrin results in loss of cell polarity and MuSC attrition but the cells remain in the niche, under the basal lamina. Although potential mechanisms for basal adhesion that do not rely on β1-integrin may exist in MuSCs, their roles are not clear (Mashinchian et al., 2018). Apical adhesion mechanisms may therefore be sufficient to provide niche localization but are insufficient to maintain cell polarity and quiescence in the absence of β1-integrin-containing adhesion receptors.

Our findings with catenin-deficient MuSCs indicate that cadherin-based adhesion is required for both niche localization and maintenance of quiescence. However, exit from the niche is not an automatic consequence of MuSC activation in the absence of injury. In addition to the example of β1-integrin, conditional mutation of the Notch-responsive transcriptional regulator RBP-J leads to MuSC activation and attrition via precocious differentiation, but the cells remain in the niche (Bjornson et al., 2012; Mourikis et al., 2012). Additionally, the Notch target collagen V signals via calcitonin receptor (CalcR) in MuSCs (Baghdadi et al., 2018). This interaction is required for MuSC quiescence but only 4% of CalcR-deficient MuSCs are present outside the basal lamina, when compared with 39% of α-cdKO MuSCs (Yamaguchi et al., 2015). Therefore, the interstitial localization of α-catenin-deficient MuSCs is likely due directly to loss of cadherin-based adhesion to the adjacent myofiber, rather than a secondary consequence of MuSC activation.

Taken together, our current and previous findings (Goel et al., 2017; Kann et al., 2022) demonstrate that cadherin-based adhesion is crucial for niche localization and preservation of MuSCs. N-cadherin is specifically required for maintenance of MuSC quiescence but not for niche localization. Quiescent MuSCs have long, heterogeneous projections that appear to serve as sensors of the stem cell niche, as they rapidly retract upon muscle injury (Kann et al., 2022; Ma et al., 2022). N-cadherin protein is enriched on projections, and N-cadherin-deficient MuSCs lack or have significantly shorter projections (Kann et al., 2022). As N-cadherin is dispensable for niche localization, we hypothesize that its major role in regulating MuSC quiescence is promoting outgrowth and/or maintenance of projections, with a minimum homeostatic projection length required for quiescence. Anchorage to the niche must be provided either by different, or by the full complement of, cadherins, as shown here by the phenotype of MuSCs lacking catenins.

Cadherin-catenin complexes are best understood as factors bridging cell-cell contact and adhesion with cytoskeletal structure and dynamic regulation of cell tension (Leckband and de Rooij, 2014; Mège and Ishiyama, 2017). Work on MuSC cellular projections suggests that the first step in injury-induced MuSC activation may be a change to the biomechanical environment of the niche (Krauss and Kann, 2023). Additionally, changes to the actin and microtubule cytoskeletons are among the earliest events visible after MuSC activation (Kann et al., 2022). How these cytoskeletal structures differ between quiescent MuSCs and MuSCs activated by injury or by partial or complete loss of cadherin-catenin-dependent adhesion is now important to address. This will require new preparations and high-resolution tools, as isolated MuSCs do not retain a physiological morphology and myofiber sarcomeres interfere with clear visualization of MuSC actin structures on single myofiber preparations (Kann et al., 2022). Our findings indicate that various types of cadherin-catenin-based adhesion are required in MuSCs for both niche localization and quiescence, and can be distinguished from the role of integrin-based adhesion, suggesting that such approaches will provide significant insight into stem cell niche function.

## MATERIALS AND METHODS

### Experimental model and subject details

#### Animals

Mice were housed and maintained in accordance with recommendations set in the Guide for the Care and Use of Laboratory Animals of the National Institutes of Health. All animal protocols used in these studies were approved by the Icahn School of Medicine at Mount Sinai Institutional Animal Care and Use Committee (IACUC).

#### Mouse breeding

To generate the *Ctnna1^flox/flox^*;*Ctnna3^flox/flox^*;*Pax7^tm1(CreERT2)Gaka/+^* line, 8- to 12-week-old homozygous *Ctnna1^flox/flox^*;*Ctnna3^flox/flox^* mice (Li et al., 2015, 2012; Vasioukhin et al., 2001) were crossed with 8- to 12-week-old *Pax7^tm1(CreERT2)Gaka^* heterozygous mice (Murphy et al., 2011) (Jackson Laboratory, 017763). The offspring were crossed until litters yielded mice homozygous for floxed alleles of αE- and αT-catenin, and heterozygous for *Pax7^tm1(CreERT2)Gaka^*. These mice were designated as α-cdKO. Littermates lacking *Pax7^tm1(CreERT2)Gaka^* were used as controls. To estimate the recombination efficiency of the system, we made the assumption that any MuSCs that were no longer detectable via Pax7 staining had undergone full recombination, i.e. lacking expression of all four α-catenin alleles and degradation of perduring protein via homeostatic turnover. Minimum recombination efficiency by this definition is 78%, the percentage of MuSCs lost in double-mutant mice compared with controls. Of the 22% of MuSCs remaining on fibers, 82% were αE-catenin immunoreactive and 78% were αT-catenin immunoreactive. We therefore conclude that 18% of the remaining 22% of MuSCs (4% of the starting population) have a complete loss of catenin protein. This yielded an estimated 82% total recombination efficiency in α-cdKO mice at 28 DPT.

To generate the *Ctnna1^flox/flox^*;*Ctnna3^flox/flox^*;*Pax7^tm1(CreERT2)Gaka/+^*;*ROSA26^LSL-tdTomato^* line, α-cdKO mice were crossed with 8- to 12-week-old homozygous *Gt(ROSA)26Sor^tm14(CAG-tdTomato)Hze^* mice purchased from Jackson Laboratory (Madisen et al., 2010) (Jackson Laboratory, 007914).

To generate the *Ctnnb1^tm2Kem/tm2Kem^*;*Jup^tm1.1Glr^*^/*tm1.1Glr*^;*Pax7^tm1(CreERT2)Gaka/+^* line, 8- to 12-week-old homozygous *Ctnnb1^tm2Kem^*;*Jup^tm1.1Glr^* mice provided by Glenn Radice (Li et al., 2011; Swope et al., 2012) (Jackson Laboratory, 004152 and 017575) were crossed with 8-12 week *Pax7^tm1(CreERT2)Gaka^* heterozygous mice (Murphy et al., 2011) (Jackson Laboratory, 017763). The offspring were crossed until litters yielded mice homozygous for floxed alleles of β- and γ-catenin, and heterozygous for *Pax7^tm1(CreERT2)Gaka^*. These mice were designated as βγ-cdKO. Littermates lacking *Pax7^tm1(CreERT2)Gaka^* were used as controls. Estimation of recombination frequency was calculated as above for α-cdKO mice. The percentage of MuSCs lost in double-mutant mice was 71% and of the remaining 29%, 53% were β-catenin-immunoreactive and 66% were γ-catenin-immunoreactive. Therefore, 34% of the remaining 29% of MuSCs (10% of the starting population) have lost β- and γ-catenin, yielding an estimated 81% total recombination efficiency in βγ-cdKO mice at 28 dpt.

To generate the *Jup^tm1.1Glr^*;*Pax7^tm1(CreERT2)Gaka/+^* line, 8- to 12-week-old *Ctnnb1^tm2Kem/tm2Kem^*;*Jup^tm1.1Glr^*^/*tm1.1Glr*^;*Pax7^tm1(CreERT2)Gaka/+^* mice were crossed with 8- to 12-week-old *Pax7^tm1(CreERT2)Gaka/+^* mice (Murphy et al., 2011) (Jackson Laboratory, 017763) until resultant litters had homozygous wild-type alleles for β-catenin and homozygous floxed alleles of γ-catenin, and were heterozygous for *Pax7^tm1(CreERT2)Gaka^.* These mice were designated as γ-cKO. Littermates lacking *Pax7^tm1(CreERT2)Gaka^* were used as controls. *Pax7^tm1(CreERT2)Gaka^; ROSA26^LSL-tdTomato^* mice were used in FACS and scRNAseq experiments as controls, and were denoted ‘Control’ in single cell RNA-sequencing experiments. Primers used for PCR genotyping are listed in Table S1.

### Method details

#### Tamoxifen administration

Adult mice (2-3 months) were injected intraperitoneally for 5 consecutive days with 125 mg/kg of tamoxifen dissolved in corn oil (Toronto Research Chemicals, T006000; ThermoFisher Scientific, 405435000). Tamoxifen supplementation in chow was used during injury experiments, as noted in the main text and figure legends (Envigo TD.130857).

#### Muscle isolation

Whole TA muscles and single myofibers from EDL muscles were isolated as previously described (Goel and Krauss, 2019). Briefly, mice were sacrificed via CO_2_ inhalation, and death was confirmed with cervical dislocation. Skin was removed from the hindlimbs from the ankle joint to the hip. Whole TA muscles were mounted in 10% w/v tragacanth gum (Alfa Aesar, A18502) and snap frozen in 2-methylbutane (Fisher Scientific, O3551-4) before being transferred to a −80°C freezer for storage. Frozen TA muscles were cryosectioned at 10 μm and collected on positively charged slides. Sections were kept at −20°C until used for immunostaining or histology.

The tendons of the EDL were cut at the ankle and knee, and the EDL was immediately incubated in plating medium (DMEM+10% horse serum+2% penicillin/streptomycin+1% HEPES) containing type I collagenase (2.6 mg/ml; Gibco, 17100-017) in a 37°C shaking water bath for 53 min, followed by trituration with a wide bore glass pipet in plating medium in a fresh 10 cm plate. Fibers were allowed to rest in a 37°C incubator for 5 min before they were collected for fixation in a final concentration of 4% paraformaldehyde in PBS for 10 min. Myofibers were washed with fresh PBS after fixation and maintained at 4°C until used for immunostaining.

#### Skeletal muscle injury

An acute barium chloride (BaCl_2_) injury model was adapted and used to study muscle regeneration (Tierney and Sacco, 2016). Fur was removed from the injection site of the anterior hindlimb with clippers and disinfected with ethanol. A 1 ml syringe equipped with a 30 g needle was used to pierce the skin nearly parallel to the ankle and inserted through the length of the TA, stopping immediately short of the knee. A repetitive process of slowly releasing 1.2% BaCl_2_ dissolved in normal saline (Thermo Scientific, 612281000) and withdrawing the needle tip slightly ensured adequate diffusion of BaCl_2_ into the muscle and minimized leakage. A total of 75 μl was injected along the length of the TA for full muscle injury. Mice were allowed to recover fully from anesthesia on a heating pad. Four weeks after the initial injury, mice were re-injured and muscles were harvested at 7 and 28 dpi.

#### BrdU pulse-chase labeling

Mice were injected intraperitoneally three times daily for 5 consecutive days with 5-bromo-2′-deoxyuridine (BrdU, Sigma B9285-1G) diluted to 30 mg/kg in PBS. Mice were harvested after a chase period of 1 week to label cells undergoing S-phase entry *in vivo*.

#### Immunofluorescence on single myofibers

After single EDL myofibers were acquired as described above, fixed myofibers were permeabilized with 0.2% TritonX-100 in PBS (PBST) for 10 min, followed by incubation with 10% goat serum in staining media for 1 h on a rotating shaker at room temperature. Primary antibodies were added and fibers were incubated overnight at 4°C on a shaker. Fibers were washed with PBS and PBST before blocking in 10% goat serum. Secondary antibodies were added to incubate for 1 h in the dark at room temperature before being washed again with PBS and PBST and mounted on positively charged slides with DAPI Fluoroshield mounting media (Abcam ab104139). Antibodies and dilutions used for immunofluorescent staining can be found in Table S2.

#### Immunofluorescence on whole muscle cross-sections

Frozen TA sections were allowed to equilibrate to room temperature and fixed in 4% paraformaldehyde in the dark for 20 min. The slides were rinsed in PBS, permeabilized in −20°C methanol for 6 min and washed with PBS. Antigen retrieval was performed using 0.01 M citric acid buffer (pH 6.0) at 90°C for 10 min. Slides were washed again in PBS, blocked in 5% BSA in PBS for 2 h, and primary antibodies were added to incubate overnight at 4°C. Slides were washed in PBS and blocked with 0.1% BSA in PBS for 30 min. Secondary antibodies were added and slides incubated for 1 h at room temperature, washed with PBS and mounted with DAPI Fluoroshield mounting media (Abcam, ab104139).

#### Picrosirius Red staining

Frozen TA sections were air dried overnight at room temperature. Before staining, sections were circled with a Pap pen (Vector Laboratories, H-4000) and allowed to dry. A hybridization chamber with a water reservoir was used as a humidity chamber and set to 56°C. The slides were added to the chamber and a disposable pipette was used to drop Bouin's fixative onto the slides and incubated for 15 min. Bouin's fixative was removed in a fume hood and slides were washed in distilled water twice before placing them into a coplin jar with 30 ml of 0.1% Sirius Red in saturated picric acid (Electron Microscopy Services, 26357-02) and incubating for 2 h on a shaker. Slides were washed in 0.5% acetic acid, dehydrated in ethanol, equilibrated in xylenes for 10 min and mounted with Cytoseal XYL (Fisher Scientific, 22-050-262).

#### TUNEL assay

The Click-iT Plus TUNEL Assay Kit for In Situ Apoptosis Detection 594 (ThermoFisher, C10618) was used to detect DNA breaks on frozen TA sections. The manufacturer protocol was used. Sections were fixed in 4% PFA for 15 min at 37°C, then washed in 1×PBS twice for 5 min. Sections were then covered and incubated with Proteinase K solution for 15 min at room temperature before washing with PBS once for 5 min. Sections were fixed a second time in 4% PFA for 5 min at 37°C, followed by two washes of PBS for 5 min and a rinse in deionized RNase-free water. A positive control was prepared by inducing DNA breaks with DNaseI solution for 30 min at room temperature before a deionized water rinse. All sections were then incubated in TdT reaction buffer for 10 min at 37°C, followed by the TdT reaction mixture at 37°C for 1 h. Sections were washed with 3% BSA and 0.1% Triton X-100 in PBS for 5 min at room temperature and rinsed with PBS after. Sections were then treated with Click-iT Plus TUNEL reaction cocktail for 30 min at 37°C in the dark, washed with 3% BSA in PBS for 5 min and rinsed with PBS. The TUNEL reaction was followed by antibody staining. Sections were blocked for 1 h at room temperature in 3% BSA in PBS and incubated with an anti-laminin antibody overnight at 4°C in the dark. Sections were washed with 3% BSA in PBS twice for 5 min and incubated with Alexa Fluor 488 anti-rabbit IgG secondary antibody for 30 min at room temperature. Sections were washed with 3% BSA in PBS twice for 5 min, dried and mounted with DAPI Fluoroshield mounting media (Abcam, ab104139).

#### Senescence-associated β-galactosidase detection

The SPiDER-βGal Detection kit (Dojindo, SG02) was used to detect senescence-associated β-galactosidase expression in whole muscle. The manufacturer's protocol was refined for use on fresh frozen TA sections. Sections were fixed in 4% PFA for 10 min in the dark at room temperature, washed once with PBS for 5 min and twice with 0.3% Triton X-100 in PBS for 5 min each. SPiDER-βGal was reconstituted in PBS and sections were incubated in the dark with SPiDER-βGal at 20 μmol/l for a1 h at 37°C. Samples were washed three times with PBS for 5 min each and incubated with WGA-488 (10 μg/ml; Invitrogen, W11261) for 30 min at room temperature, before washing again three times with PBS for 5 min each and mounted with DAPI Fluoroshield mounting media (Abcam, ab104139).

#### Isolation of MuSCs

Single cell suspensions from mouse hindlimb whole muscle were acquired with modifications from a protocol described previously (Gromova et al., 2015). After euthanasia, skin was removed from both hindlimbs, and whole hindlimbs were removed to the hip and placed on ice in PBS. Muscles were excised with care to remove fat, tendons, blood vessels, nerves and bones. Muscles were minced with a sterile razor blade and transferred into digestion medium containing collagenase II and dispase II (Fisher Scientific, 17-101-015; 17-105-041) for enzymatic dissociation of mononuclear cells from muscle fibers in a rotating water bath at 37°C. A 10 ml syringe with a 20 g needle was used further release mononuclear cells into suspension before being passed through 40 μm cell strainers.

Cells were pelleted at 300 ***g*** for 5 min before washing with ice-cold Hanks' Balanced Salt Solution without magnesium and calcium supplemented with 2% FBS and 1 mM EDTA (HBSS+), pelleting again and resuspending in 4 ml of HBSS+. Samples were kept on ice and counterstained with SYTOX Blue before FACS isolation of MuSCs. Each sample was pooled from two littermate mice of the same genotype with two samples sequenced per genotype. A Sony MA900 equipped with a 100 μm nozzle and 20 psi pressure was used for FACS isolation.

#### Single cell RNA sequencing analysis processing

Sequenced fastq files were aligned, filtered, barcoded and UMI counted using Cell Ranger Chromium Single Cell RNA-seq version 7.1.0, by 10X Genomics with Cell Ranger, GRCm38 database (version 2020-A) as the mouse genome reference. Each dataset was filtered to retain cells with ≥1000 UMIs, ≥400 genes expressed and <15% of the reads mapping to the mitochondrial genome. UMI counts were then normalized so that each cell had a total of 10,000 UMIs across all genes and these normalized counts were log transformed with a pseudocount of 1 using the “LogNormalize” function in the Seurat package. The top 2000 most highly variable genes were identified using the “vst” selection method of “FindVariableFeatures” function and counts were scaled using the “ScaleData” function. Datasets were processed using the Seurat package (version 4.0.3) (Hao et al., 2021).

Principal component analysis was performed using the top 2000 highly variable features (“RunPCA” function) and the top 30 principal components were used in the downstream analysis. Datasets for each patient were integrated by using the “RunHarmony” function in the harmony package (version 0.1.0) (Korsunsky et al., 2019). K-Nearest Neighbor graphs were obtained by using the “FindNeighbors” function, whereas the UMAPs were obtained by the “RunUMAP” function. The Louvain algorithm was used to cluster cells based on expression similarity at 0.2 resolution.

Differential markers for each cluster were identified using the Wilcox test (“FindAllMarkers” function) with adjusted *P*-value <0.05 and absolute log2 fold change >0.25, and 1000 cells per cluster were randomly picked to represent each cluster. Top two significant markers in terms of log2 fold change for all clusters were plotted using Seurat DotPlot command. The top upregulated genes and curated genes from the literature were used to assign cell types to the clusters. Unicell Deconconvolve was used to annotate the cells using the log normalized counts of the integrated dataset (version 0.0.3) (Charytonowicz et al., 2023). Prediction values for each cell type are merged with the original Seurat object. UMAPs of prediction value for skeletal and striated muscle are generated using the Seurat Featureplot function (version 4.0.3) (Hao et al., 2021).

*P* values for cell proportions within clusters were calculated using a logistic regression method. The lme4 package was used to fit data to a logistic regression model (version 1.1-27) (Bates et al., 2015). The odds ratio and *P*-value of seeing this ratio between conditions was calculated using the emmeans package (version 1.6.2) (Lenth et al., 2023).

### Quantification and statistical analysis

#### Single myofiber imaging and quantification

Images were collected using 100×/1.40 oil objectives with up to an additional 2× digital zoom on a Leica DMI SP8 inverted confocal microscope equipped with Leica Application Suite. Line averaging was used to improve signal-to-noise ratio in representative images as follows: line average-3, frame average-2. Images were exported using the native Leica Application Suite as TIFFs and imported to ImageJ for post-imaging analysis with adjustment of brightness and contrast only. For quantification of cell numbers and presence of catenin protein, as indicated by immunofluorescent signal, at least 10 fibers were analyzed per mouse with *n*≥4 per genotype, as noted in figure legends.

#### Whole-muscle cross-section imaging and quantification

Wide-field images were collected using a 20×/0.75 air objective on a Zeiss AxioImager Z2 equipped with Zen Blue Software. Images were exported using the native Zeiss Application Suite as .czi files or TIFFs and imported to ImageJ for post-imaging analysis with adjustment of brightness and contrast only.

For quantification of cell numbers during homeostasis and regeneration, 10 random fields of view were taken at 20× magnification per mouse with *n*≥3 per genotype, as noted in figure legends. For quantification of cell localization, cell expression of activation markers and BrdU incorporation, a minimum of 30 cells were counted per animal with *n*≥3 per genotype, as noted in figure legends. Picrosirius Red staining was quantified using ImageJ where images were segmented by red-stained collagen and measuring percent coverage by the segmented area. Myofiber minimum feret diameter was quantified using the ImageJ package MuscleJ (Mayeuf-Louchart et al., 2018).

#### Statistical analyses

All experiments, excluding FACS isolation and single cell RNA-sequencing experiments, were performed using mice with *n*≥3 per genotype as specified in figure legends. Mean, standard error of mean (s.e.m.), 95% confidence intervals, unpaired two-tailed Student's *t*-tests and Mann–Whitney tests were calculated with Prism GraphPad Software. All graphs were generated with Prism GraphPad Software or R. Fig. S1 shares control and βγ-cdKO data from Fig. 1.

## Supplementary Material



10.1242/develop.202387_sup1 Supplementary information

## The people behind the papers

This article has an associated ‘The people behind the papers' interview with some of the authors.
