# Supplementary material for: Cadherin-dependent adhesion is required for muscle stem cell niche anchorage and maintenance
Source: Development. 2024 Apr 4;151(7):dev202387. doi: 10.1242/dev.202387 (PMC11057819; doi:10.1242/dev.202387)
Supplement: Supplementary information [file develop-151-202387-s1.pdf]

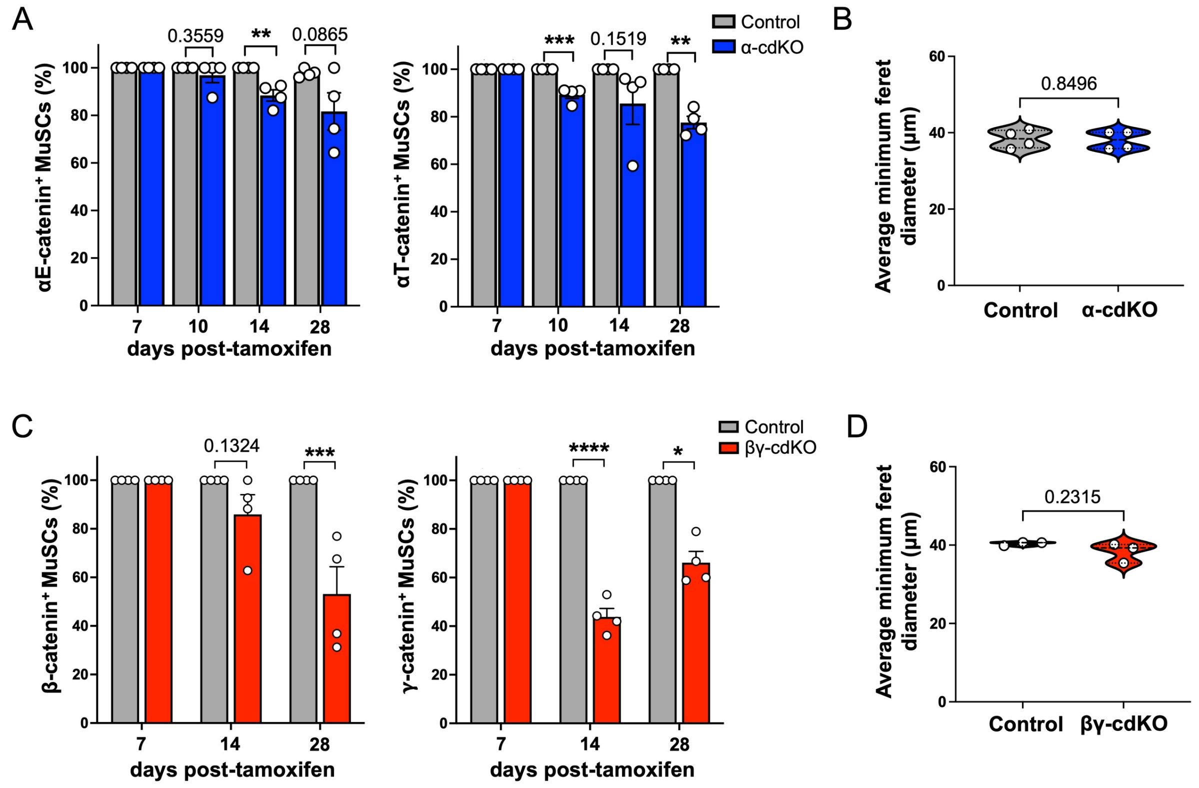

Fig. S1. Quantification of catenin^{+} MuSCs over time after Tamoxifen treatment

(A) Immunostaining of single myofibers as shown in Fig. 1D,E was used to assess the presence of αE- and αT-catenin protein over a 28 day time course post-TMX.

(B) At 28 DPT, TA muscle sections from control and α-cdKO mice were immunostained with membrane marker laminin and myofiber size was quantified by minimum feret diameter, displayed as average myofiber size per animal.

(C) Immunostaining of single myofibers as shown in Fig. 1J,K was used to assess the presence of β- and γ-catenin protein over a 28 day time course post-TMX.

(D) At 28 DPT, TA muscle sections from control and βγ-cdKO mice were immunostained with membrane marker laminin and myofiber size was quantified by minimum feret diameter displayed as average myofiber size per animal.

Each data point represents the average from at least ten myofibers (EDL single myofibers) or ten fields (TA muscle sections) from each animal. Data represent n≥3 per genotype per timepoint and represented as mean ± S.E.M with comparisons by two-tailed unpaired Student's t-test, unless otherwise noted. * = $p < 0.05$, ** = $p < 0.01$, *** = $p < 0.001$.

Development • Supplementary information

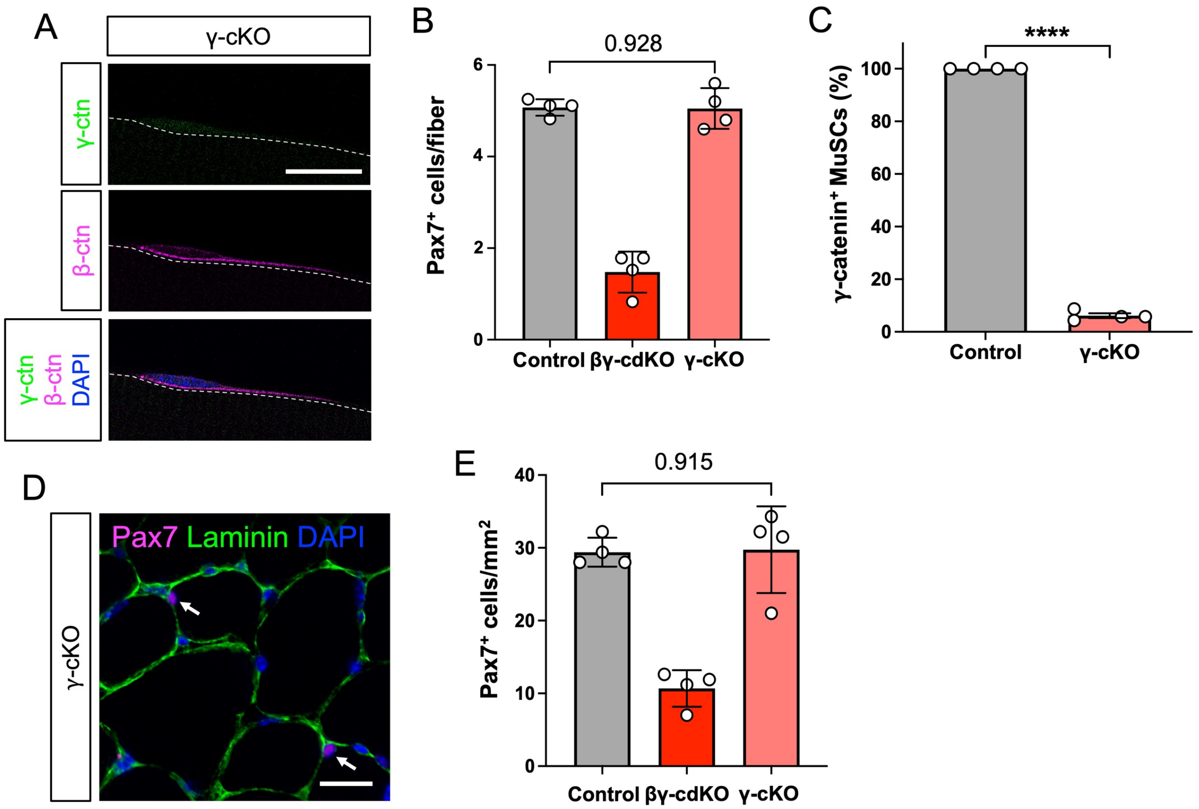

Fig. S2. Genetic removal of γ-catenin does not lead to MuSC attrition or alter β-catenin localization

(A-C) Immunostaining of single myofibers from 28 DPT γ-cKO mice (γ-catenin, green; β-catenin, magenta, DAPI, blue) was used to assess loss of γ- and/or β-catenin protein (A; see Fig. 1J,K for examples of control cells), MuSC attrition (B), and percentage of remaining MuSCs with γ-catenin protein (C). Scale bar: 10 μm.

(D-E) TA muscle sections from 28 DPT γ-cKO mice were immunostained (Pax7, magenta; laminin, green; DAPI, blue) and Pax7-expressing MuSCs were labeled (D) and quantified (E). Scale bar: 25 μm.

Each data point represents the average from at least ten myofibers (EDL single myofibers) or ten fields (TA muscle sections) from each animal. Data represent n=4 per genotype per timepoint and represented as mean ± S.E.M with comparisons by two-tailed unpaired Student's t-test. **** = $p < 0.0001$.

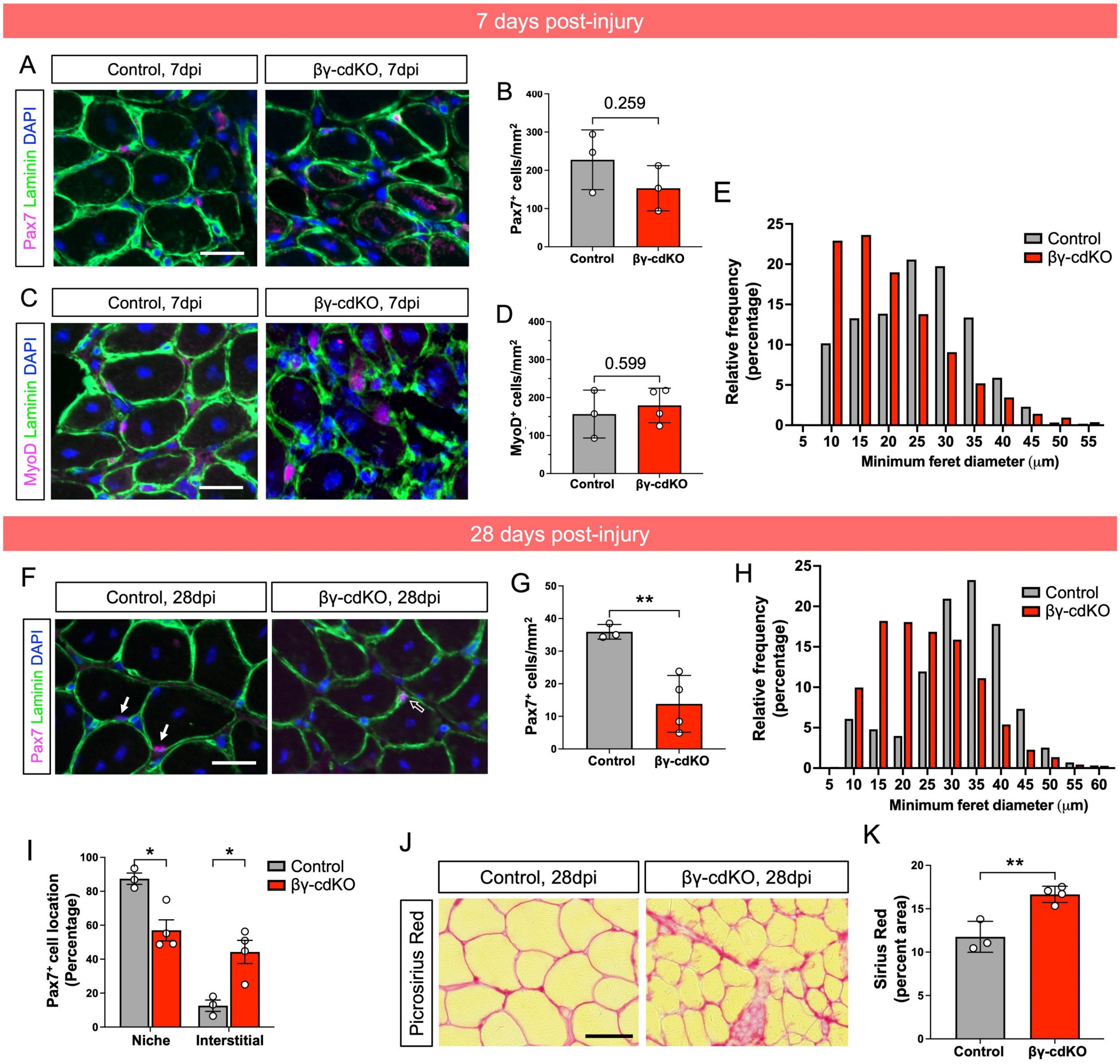

Fig. S3. βγ-cdKO mice have modest defects in muscle regeneration

(A-E) At 7dpi, TA muscle sections from control and βγ-cdKO mice were immunostained (Pax7 or MyoD, magenta; laminin, green; DAPI, blue) for $Pax7^{+}$ (A,B) and $MyoD^{+}$ (C,D) cells to assess MuSC and myogenic progenitor numbers during early regeneration. Myofiber size was quantified by minimum feret diameter in (E).

(F-K) At 28dpi, TA muscle sections from control and βγ-cdKO mice were immunostained (Pax7, magenta; laminin, green; DAPI, blue) (F) to assess MuSC numbers (G). Myofiber size was quantified by minimum feret diameter in (H). $Pax7^{+}$ cell location was scored in (I). Sections of TA muscle from control and βγ-cdKO mice at 28dpi with Picrosirius Red (J) to quantify collagen deposition after injury (K).

Scale bars: 25 µm (A,C,F). Solid arrow denotes cell under the basal lamina; open arrow denotes interstitial cell. Each data point represents the average from ten fields from each animal. All data represent n≥3 per genotype and represented as mean ± S.E.M with comparisons by two-tailed unpaired Student's t-test. * = $p < 0.05$, ** = $p < 0.01$.

Development • Supplementary information

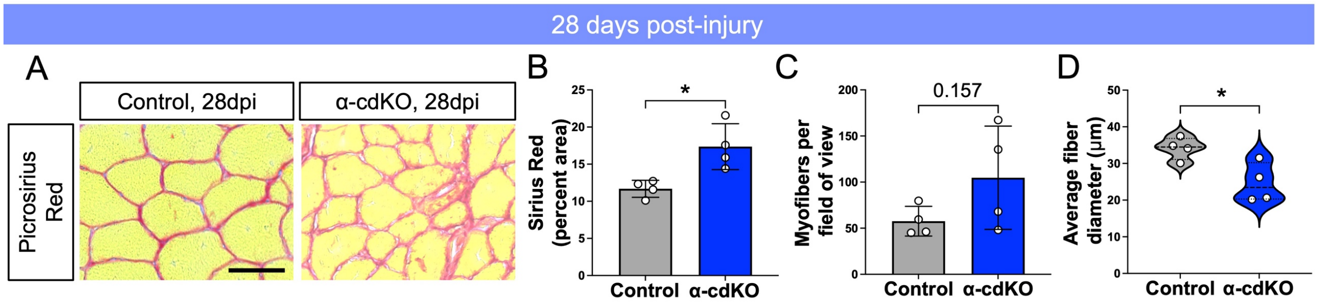

Fig. S4. α-cdKO mice have modest defects in muscle regeneration

(A-B) Sections of TA muscle from control and α-cdKO mice at 28dpi with Picrosirius Red (A) to quantify collagen deposition after injury (B). Scale bar: 25 μm.

(C-D) Average number of myofibers per field of view at 20x magnification was quantified (C), as was the average fiber diameter (D) from control and α-cdKO mice at 28dpi. Both correlated with the significant decrease in average myofiber size in Figure 2J,K.

Each data point represents the average from ten fields (TA muscle sections) from each animal. Data represent n=4 per genotype per timepoint and represented as mean ± S.E.M with comparisons by two-tailed unpaired Student's t-test. * = $p < 0.05$.

Development • Supplementary information

A

	Control-1	Control-2	α-cdKO-1	α-cdKO-2
Estimated number of cells	5,853	6,319	10,649	8,433
Fraction reads in cells	96.9%	94.9%	95.6%	97.6%
Mean reads per cell	74,976	89,685	47,060	65,561
Median genes per cell	1,724	1,623	1,527	1,726
Median UMI counts per cell	7,485	7,282	6,186	7,574

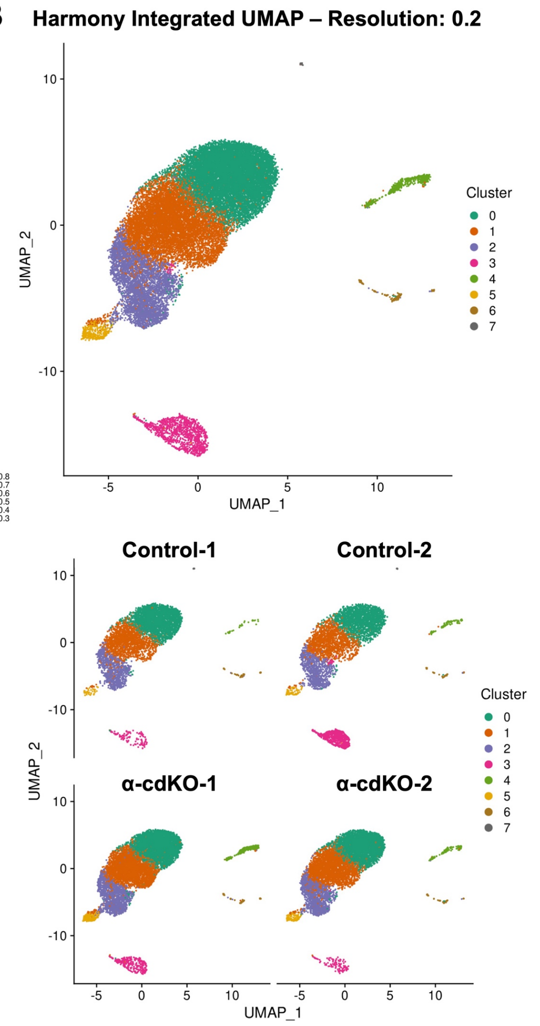

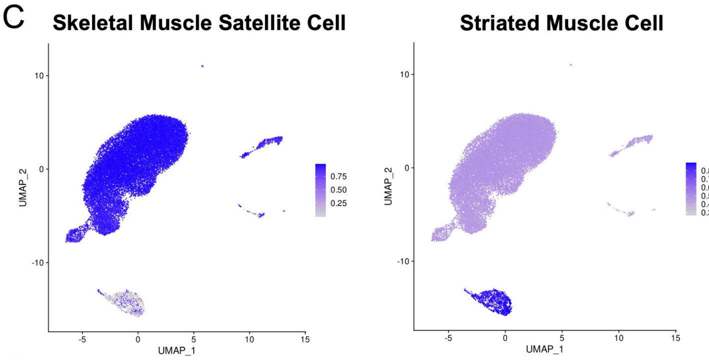

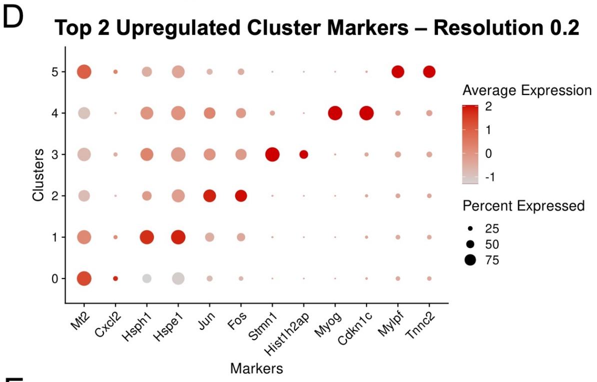

E

Cluster	Annotation	Top differentially expressed genes (DEGs)
0*	Activation-2	Sdc4, Rhoj, Cxcl1, Myod1, Cxcl2
1*	Activation-1	Bag3, Cacybp, Maff, Serpinh1, Jund
2*	Quiescent	Jun, Fos, Hes1, Gas1, Egr1
3	Cycling	Stmn1, Top2a, Mki67, Lgals1, Smc4
4	Differentiated	Myog, Mymk, Cdkn1c, Actc1, Chchd10
5	Mature myocyte	Tnnc2, Mylpf, Tnnt3, Ckm, Tnni2

Fig. S5. Quality control during cell purification and genomic analyses

(A) Single cell RNA sequencing quality control of tdTomato$^+$ cells from control and α-cdKO at 14 DPT was assessed using CellRanger.

(B) tdTomato$^+$ cells from control and α-cdKO at 14 DPT were integrated using Harmony and unsupervised clustering yielded a UMAP with eight cell clusters, none of which were unique to one genotype.

(C) UniCell Deconvolve cell type predictions score for MuSC (left) and striated muscle cell types (right). A much smaller proportion of cells were classified as mature myocytes ('Striated Muscle Cell') compared to MuSCs ('Skeletal Muscle Satellite Cell').

(D-E) Differentially upregulated genes per cluster (D) indicate isolation-induced stress response in some clusters, but used in conjunction with other differentially expressed genes per cluster, allowed author annotation of cluster identities (E).

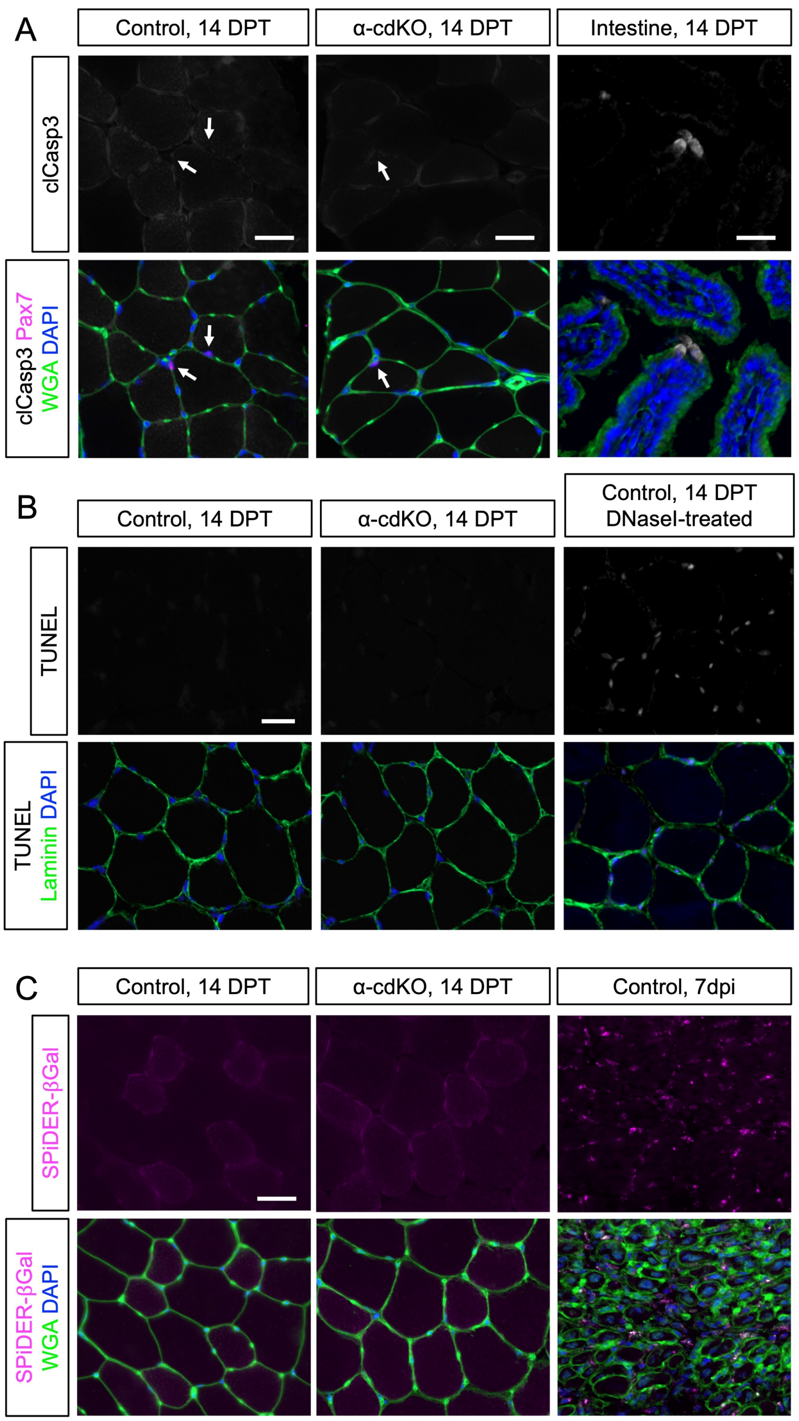

Fig. S6. Lack of evidence for apoptosis or senescence of α-cdKO MuSCs

(A) Immunostaining of sections through TA muscle of control and α-cdKO mice at 14 DPT for Pax7 (magenta), cleaved Caspase-3 (grey), WGA (green), and DAPI (blue). Intestine used as a positive control. Arrows denote location of $Pax7^+$ cells. No evidence of cleaved Caspase-3 was

Development • Supplementary information

found in muscle sections was found. Scale bars: 25µm for muscle sections, 100µm for intestine section.

(B) TUNEL detection (grey) with immunostaining of sections through TA muscle of control and α-cdKO mice at 14 DPT for Laminin (green), and DAPI (blue). Control TA muscle section treated with DNaseI used as a positive control. No evidence of TUNEL detection of DNA breakage was found. Scale bar: 25µm.

(C) Senescence-associated β-galactosidase detection via immunofluorescent SPiDER-βgal (magenta) with immunostaining of sections through TA muscle of control and α-cdKO mice at 14 DPT for WGA (green), and DAPI (blue). No evidence of senescence-associated β-galactosidase was detected. A TA muscle section at 7dpi was used as a positive control. Scale bar: 25µm.

All images represent n=3 per genotype.

Development • Supplementary information

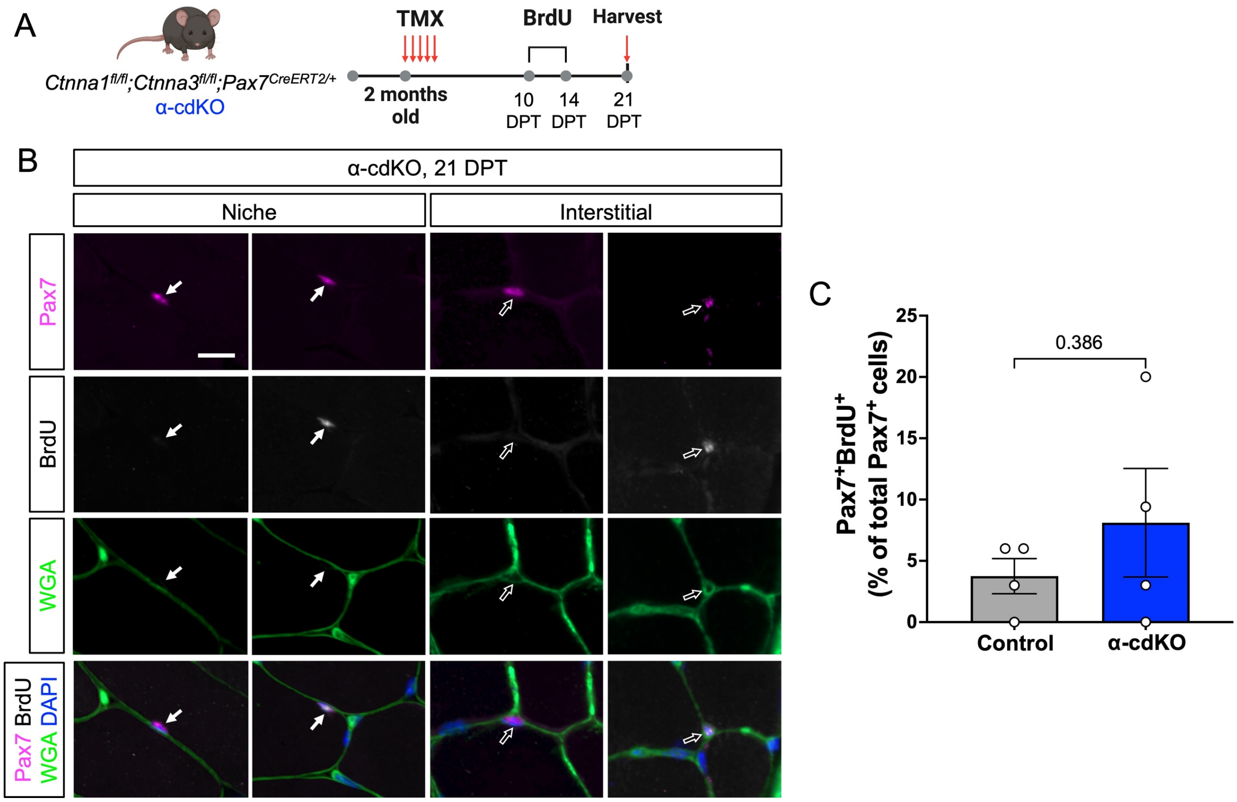

Fig. S7. Remaining Pax7$^+$ α-cdKO MuSCs are not more likely to incorporate BrdU than controls at 21 days post-tamoxifen

(A) Experimental scheme of BrdU pulse-chase experiment to confirm cell cycle progression in MuSCs from control and α-cdKO mice. TA muscles were harvested and snap frozen at 21 DPT for immunofluorescence analysis. Made with BioRender.

(B-C) Immunostaining of sections through TA muscle from α-cdKO mice 21 DPT (Pax7, magenta; BrdU, grey; WGA, green; and DAPI, blue) (B) showed that there was no significant differences in BrdU incorporation among Pax7$^+$ cells (C). Solid arrow denotes cell under the basal lamina; open arrow denotes interstitial cell. Scale bar: 10μm.

Each data point represents the average from ten fields (TA muscle sections) from each animal. All data represent n=4 per genotype and represented as mean ± S.E.M with comparisons by two-tailed unpaired Student's t-test.

Table S1. Primers used for genotyping

Name	Sequence (5'-3')
Pax7CreERT2 13984 Common Forward	GCT GCT GTT GAT TAC CTG GC
Pax7CreERT2 139845 Wild Type Reverse	CTG CAC TGA GAC AGG ACC G
Pax7CreERT2 139846 Mutant Reverse	CAA AAG ACG GCA ATA TGG TG
β-catenin flox oIMR1512F	AAG GTA GAG TGA TGA AAG TTG TT
β-catenin flox oIMR1513R	CAC CAT GTC CTC TGT CTA TTC
γ-catenin flox For	AAG AAA TAC CCA CGG CTC CT
γ-catenin flox Rev	GCT CCA GGG AGA AAC AGA CA
αE-catenin For	CAT TTC TGT CAC CCC CAA AGA CAC
αE-catenin Rev	GCA AAA TGA TCC AGC GTC CTG GG
αT-catenin For	CCA CCC CTG ATA TGA CCT GTA G
αT-catenin Rev	TCC CCA GGA ATC AAG TCG TT
Rosa26-tdTomato MtF	CTGTTCCTGTACGGCATGG
Rosa26-tdTomato MtR	GGCATTAAAGCAGCGTATCC
Rosa26-tdTomato WtF	AAGGGAGCTGCAGTGGAGTA
Rosa26-tdTomato WtR	CCGAAAATCTGTGGGAAGTC

Development • Supplementary information

Table S2. Antibodies and dilutions for immunofluorescence

Antibody	Dilution	Source
Mouse anti-Pax7	1:100	DSHB (Pax7-c)
Rabbit anti-Caveolin-1	1:200	Abcam (ab2910)
Mouse anti-MyoD	1:100	BD Biosciences (554130)
Mouse anti-Myogenin	1:100	DSHB (F5D)
Rabbit anti-Laminin	1:100	Sigma (L9393)
Mouse anti-β-catenin	1:200	BD Biosciences (610154)
Rabbit anti-γ-catenin	1:75	Cell Signaling Technology (2309S)
Rabbit anti-αT-catenin	1:100	Proteintech (13974-1-AP)
Rabbit anti-αE-catenin	1:100	Invitrogen (71-1200)
Rabbit anti-M-cadherin	1:200	Cell Signaling Technology (40491S)
Rat anti-BrdU	1:300	Abcam (ab6326)
Rabbit anti-Ki67	1:500	Abcam (ab15580)
Rabbit anti-cleaved Caspase 3 (Asp175)	1:300	Cell Signaling Technology (9664)
Anti-WGA-488 conjugated	10 µg/ml	Invitrogen (W11261)
Alexa Fluor 647 anti-Mouse IgG1	1:500	ThermoFisher (A-21240)
Alexa Fluor 647 anti-Rabbit IgG	1:500	ThermoFisher (A-21245)
Alexa Fluor 488 anti-Rabbit IgG	1:500	ThermoFisher (A-11008)
Alexa Fluor 488 anti-Mouse IgG1	1:500	ThermoFisher (A-21121)
Alexa Fluor 568 anti-Rat IgG	1:500	ThermoFisher (A-11077)
Alexa Fluor 568 anti-Rabbit IgG	1:500	ThermoFisher (A-11011)

Development • Supplementary information
