# Supplementary material for: Cadherin-dependent adhesion is required for muscle stem cell niche anchorage and maintenance
Source: Development. 2024 Apr 4;151(7):dev202387. doi: 10.1242/dev.202387 (PMC11057819; doi:10.1242/dev.202387)
Supplement: Supplementary file 1 [file develop-151-202387_review_history.pdf]

# Cadherin-dependent adhesion is required for muscle stem cell niche anchorage and maintenance

Margaret Hung, Hsiao-Fan Lo, Aviva G. Beckmann, Deniz Demircioglu, Gargi Damle, Dan Hasson, Glenn L. Radice and Robert S. Krauss
DOI: 10.1242/dev.202387

**Editor**: Benoit Bruneau

**Review timeline**
Original submission: 29 September 2023
Editorial decision: 6 November 2023
First revision received: 2 February 2024
Accepted: 25 February 2024

---

**Original submission**

First decision letter

MS ID#: DEVELOP/2023/202387

MS TITLE: Cadherin-dependent adhesion is required for muscle stem cell niche anchorage and maintenance

AUTHORS: Margaret Hung, Hsiao-Fan Lo, Aviva G. Beckmann, Deniz Demircioglu, Gargi Damle, Dan Hasson, Glenn L. Radice, and Robert S. Krauss

I have now received all the referee reports on the above manuscript, and have reached a decision. The referees' comments are appended below, or you can access them online: please go to BenchPress and click on the 'Manuscripts with Decisions' queue in the Author Area.

The overall evaluation is positive and we would like to publish a revised manuscript in Development, provided that the referees' comments can be satisfactorily addressed. Please attend to all of the reviewers' comments in your revised manuscript and detail them in your point-by-point response. If you do not agree with any of their criticisms or suggestions explain clearly why this is so. If it would be helpful, you are welcome to contact us to discuss your revision in greater detail. Please send us a point-by-point response indicating your plans for addressing the referees' comments, and we will look over this and provide further guidance.

Reviewer 1

*Advance summary and potential significance to field*

This is a very interesting study that addresses the nature of adhesion interactions between an adult tissue specific stem cell and its niche. These adhesion interactions are important to stabilize proximity of stem cells to sources of biochemical and/or biomechanical signaling mechanisms.

In muscle stem cells, adhesion interactions are mediated in part by cadherin-based adhesion interactions, but the importance of these interactions to maintain quiescence had not been demonstrated, potentially in part due to redundancy between different cadherins that are expressed at different levels.

© 2024. Published by The Company of Biologists under the terms of the Creative Commons Attribution License (https://creativecommons.org/licenses/by/4.0/).

Therefore, to ask broadly whether cadherin interactions mediate MuSC quiescence the investigators used genetic tools in the mouse to remove the catenins, which are cytoplasmic proteins that are essential for cadherin dependent adhesion. The investigators show that catenin-deficient MuSCs break quiescence, exit the niche and are progressively depleted in consequence to precocious differentiation and fusion to myofiber.

The study addresses a timely and novel topic in stem cell biology, particularly with respect to stem cell niche interactions. The creative use of genetic tools in the mouse to remove catenins lead to a clean demonstration of the importance of cadherin-based interactions to anchor MuSCs in their niche. The presented data is high quality and the manuscript is well written.

*Comments for the author*

I have only a number of concerns that should be addressed by the authors to strengthen the manuscript:

1. Validation of genetic tools: the extent to which floxed alleles undergo recombination should be validated and reported in the manuscript. The addition of this data is important to interpret the findings. The current analysis at 28d after a large proportion of MuSCs have disappeared, may reflect the MuSCs that escape recombination (as the authors suggest) rather than report on the MuSCs that have recombined. This concerns is potentially addressed at short timepoints after tmx administration before depletion of the MuSC pool, using qPCR and primers against Ctnna1, Ctnna3, Ctnnb1 and Jup; and/or with available antibodies.

2. The authors present data that a-cdKO and bg-cdKO mice display incomplete regeneration and do not efficiently reoccupy the niche, but the conclusions should be supported by additional data.

a. It is not clear whether the observation that skeletal muscle regeneration is delayed is in consequence to depletion of the stem cell pool or due to MuSCs that are defective for catenins. This is potentially addressed with similar acute ctx injury experiments, but at timepoints before the MuSC pool is depleted, eg/ day 7.
b. The conclusions would be supported by reporting on the identity of MuSCs reoccupying the niche as wt or mutant. For example, if a greater proportion of MuSCs that are present 28d after CTX injury still express the floxed allele stronger conclusions can be made. This concern is potentially addressed using available antibodies.

3. In Figure 1, please report the timepoints of the IF analyses.

4. Figure 2, Figure 5A,D. For completeness please include scale bars on all images when wt vs mutant muscle is compared. (the reader would otherwise need to assume that the scale between images is the same)

The following minor concerns and/or suggestions can also be addressed by the authors, but I consider these non-essential revisions.

1. The authors likely did not attempt to support their conclusions with ex vivo analyses of wt vs mutant MuSCs because these analyses would eliminate the important niche interactions, but the authors might consider the following:
a. Ex vivo analysis of MuSCs in culture would remove the MuSCs from their adhesion interactions. With the genetic tools already in hand, the authors could use analyses ex vivo to support their conclusions that the catenins are important for mediating adhesion interactions and not signaling pathways described on pg 6 line 160-161.
b. Ex vivo analysis of wt vs mutant MuSCs present on cultured single EDL myofibres (partial niche with the myofiber intact) would inform on ability of MuSCs to differentiate and/or self-renew, potentially addressed with antibodies against PAX7 and/or myogenin.

2. Figure 4AB, do MuSCs that lose projections in consequence to a-cdKO also lose the localization or expression of M-cadherin?

© 2024. Published by The Company of Biologists under the terms of the Creative Commons Attribution License (https://creativecommons.org/licenses/by/4.0/).

3. Given the model that MuSCs that do not express catenins escape quiescence proliferate, differentiate and fuse with the myofiber, it would be interesting to report on potential for myonuclear accretion (myonuclei per myofibre) and/or myofibre size (myofibre CSA) in uninjured mice and/or the contralateral uninjured hindlimb. (If extensive proliferation of activated MuSCs does not occur, one might not expect a biologically significant result and so this interest is not considered an essential revision to the manuscript.)

4. Pg 5, line 142-143. Suggestion to report these data graphically.

Reviewer 2

*Advance summary and potential significance to field*

This work follows up on prior results from the Krauss lab which showed that satellite cell-specific loss of m-cadherin (the adhesion molecule which is most highly expressed and has historically been most strongly associated with satellite cells) did not cause significant changes in cell localization or activity either during quiescence or following activation, while deletion of n-cadherin led to loss of quiescence in the absence of injury. While this phenotype was exacerbated by deletion of both m- and n-cadherin, the affected cells nevertheless remained localized in the sublaminar niche, suggesting that niche adhesion and activation may be separable events. In the current manuscript, Hung et al. elegantly demonstrate that this is indeed the case, and in addition show that when satellite cells lose the ability to form cadherin/catenin-dependent adhesions to the myofiber they will differentiate and fuse with the fiber, thus depleting the quiescent satellite cell pool.

In addition to m- and n-cadherins, at least two other cadherins are present at low levels in satellite cells, thus the group chose instead to delete the intracellular mediators of cadherin signaling, separately deleting two pairs of redundant catenins (β- and γ- catenin, and αE- and αT-catenin) from satellite cells. In both experiments they observed a gradual loss of quiescent satellite cells over the 4 weeks following tamoxifen injection- in conjunction with a decrease in sublaminar Pax7+ cells, an accumulation of Pax7+MyoD+ sublaminar cells as well as MyoD+Ki67+ myoblasts in the interstitial space. The remaining satellite cells, when challenged by barium chloride injury, were blunted in their regeneration capacity, although numerically they should have been sufficient. Further, following injury, the number of sublaminar satellite cells generated by self-renewal was equivalent to the diminished population prior to injury rather than the original complement prior to tamixofen. The 'lost' cells were instead located in the interstitial space, leading to the conclusion that cadherin/catenin-dependent adhesion is necessary to reoccupy the niche.

Molecular analysis of satellite cell morphology and membrane adhesions showed that catenin dKO satellite cells lacked or had diminished membrane protrusions, which the Krauss lab has characterized as a property of quiescent satellite cells; expression and/or localization of m-cadherin is also lost in a significant fraction of sublaminar satellite cells in the catenin DKO.

In addition to further defining the role of individual molecules and their interactions in establishment and maintenance of satellite cell quiescence, these studies provide significant new understanding of the redundant and nonredundant roles of cadherins and catenins in critical aspects of satellite cell biology. In particular, they suggest that N-cadherin is specifically required for maintaining quiescence, distinct from the requirement for cadherins in general in maintaining niche adhesion. This may be due to its localization at and activity in the long cellular extensions this lab and others have shown to be associated with maintaining quiescence and mediating activation. The paper is straightforward and well written, and I particularly appreciate the thoughtful treatment of additional aspects of satellite cell biology which loss of catenins in satellite cells could potentially influence and the discussion of why these are unlikely to directly affect the observed phenotype.

*Comments for the author*

While it is not absolutely necessary, if it is feasible it would be nice for the authors to follow up on their suggestion that the sublaminar, unactivated satellite cells which are retained 4 weeks after tamoxifen are examples of incomplete/insufficient deletion of the targeted catenin pairs.

© 2024. Published by The Company of Biologists under the terms of the Creative Commons Attribution License (https://creativecommons.org/licenses/by/4.0/). 

Reviewer 3

*Advance summary and potential significance to field*

In this study, Hung and colleagues generated murine models of muscle stem cell specific, conditional knockouts, of catenin encoding genes to study cadherin-deficiency in homeostasis and regeneration. Using a combination of in vivo, ex vivo and single cell RNA-sequencing approaches, the authors describe a cadherin-dependant mechanism for satellite cell adhesion and maintenance of quiescence within the niche. This study is original and sheds light on important mechanisms for satellite cell niche localisation and regenerative capacity. It also represents a considerable body of work, not least because of the use of multiple knockout lines of mice. Additionally, the authors carried out a characterisation of the fate of cadherin-deficient MuSC and revealed that satellite cell attrition occurs via precocious differentiation, re-entry to the niche and fusion to myofibres. Altogether, this article brings interesting observations that will benefit the muscle and stem cell communities.

*Comments for the author*

The flow of the study is very logical and does not suffer from any major technical issue, only a few minor comments are listed below:

In Fig.1 and 2, the authors described a decline in Pax7+ MuSC numbers in alpha-cdKO and betagamma-cdKO mice. They confirmed that satellite cell attrition was not the consequence of apoptosis using TUNEL assay and cleaved-caspase 3 staining in Fig.S5. However, could a proportion of these cells be senescent and therefore, not contributing to muscle regeneration? That could explain the regeneration deficit and CSA decrease in catenin-deficient mice.

In Fig.3, the authors performed single cell RNA-sequencing of lineage traced Pax7Cre-tdT mice in controls and alpha-cdKO mice. Have the authors considered performing histology analysis of the skeletal muscles of these mice at 14 or 28 days post-tamoxifen? As a majority of quiescent satellite cells are lost due to precocious differentiation and fusion to existing myofibres, this could also be confirmed by in an increased number of Tomato+ fibres in alpha-cdKO mice. Some simple histology analyses in the knockout model would provide strongly confirmatory data for the authors model of the fate of satellite cells released from quiescence.

In Fig.3D, the table show the percentage of cells belonging to the control or alpha-cdKO sample, present in each of the defined clusters, based on the Harmony integrated UMAP. The authors are claiming a significant increase in the number of cycling and differentiated cells in the alpha-cdKO sample, however, in Fig.S4A the number of cells in alpha-cdKO samples (alpha--cdKO-1, alpha--cdKO-2) is almost twice as the ones in control samples (Control-1, Control-2). Are the authors normalising to the total number of cells? While it is expected and supported by the authors immuno-histochemistry data that the number of differentiated cells are increased in alpha-cdKO samples, it is surprising that the number of alpha-cdKO cells are decreased as compared to the controls in the cluster 5, corresponding to mature myocytes. Have the authors considered performing differential expression analysis between control and alpha-cdKO groups to characterise a potential shift in cell identity or gene compensation mechanisms?

---

**First revision**

Author response to reviewers' comments

MS ID#: DEVELOP/2023/202387
MS TITLE: Cadherin-dependent adhesion is required for muscle stem cell niche anchorage and maintenance

© 2024. Published by The Company of Biologists under the terms of the Creative Commons Attribution License (https://creativecommons.org/licenses/by/4.0/).

AUTHORS: Margaret Hung, Hsiao-Fan Lo, Aviva G. Beckmann, Deniz Demircioglu, Gargi Damle, Dan Hasson, Glenn L. Radice, and Robert S. Krauss

We are grateful to the reviewers for their kind review and helpful suggestions to strengthen our manuscript ahead of publication. Responses to specific comments follow below.

Reviewer 1 Advance summary and potential significance to field

This is a very interesting study that addresses the nature of adhesion interactions between an adult tissue specific stem cell and its niche. These adhesion interactions are important to stabilize proximity of stem cells to sources of biochemical and/or biomechanical signaling mechanisms.

In muscle stem cells, adhesion interactions are mediated in part by cadherin-based adhesion interactions, but the importance of these interactions to maintain quiescence had not been demonstrated, potentially in part due to redundancy between different cadherins that are expressed at different levels.

Therefore, to ask broadly whether cadherin interactions mediate MuSC quiescence, the investigators used genetic tools in the mouse to remove the catenins, which are cytoplasmic proteins that are essential for cadherin dependent adhesion. The investigators show that catenin-deficient MuSCs break quiescence, exit the niche and are progressively depleted in consequence to precocious differentiation and fusion to myofiber.

The study addresses a timely and novel topic in stem cell biology, particularly with respect to stem cell niche interactions. The creative use of genetic tools in the mouse to remove catenins lead to a clean demonstration of the importance of cadherin-based interactions to anchor MuSCs in their niche. The presented data is high quality and the manuscript is well written.

We thank the reviewer for their supportive comments.

Reviewer 1 Comments for the author
I have only a number of concerns that should be addressed by the authors to strengthen the manuscript:

1. Validation of genetic tools: the extent to which floxed alleles undergo recombination should be validated and reported in the manuscript. The addition of this data is important to interpret the findings. The current analysis at 28d, after a large proportion of MuSCs have disappeared, may reflect the MuSCs that escape recombination (as the authors suggest) rather than report on the MuSCs that have recombined. This concerns is potentially addressed at short timepoints after tmx administration before depletion of the MuSC pool, using qPCR and primers against Ctnna1, Ctnna3, Ctnnb1 and Jup; and/or with available antibodies.

We thank the reviewer for this suggestion and have added new experimental results to the paper in response. First, we performed IF analysis on a-cdKO and b/g-cdKO myofibers at days 7, 10, and 14 post-tamoxifen (TMX). These results confirm the data we reported in the original manuscript in analyses at day 28: at all time points post-TMX, a majority of remaining MuSCs are positive for one or the other catenin protein in both lines. Stated another way, MuSCs that lose both catenin proteins in either a-cdKO and b/g-cdKO mice do not accumulate substantially, even at earlier time points. All these data are presented graphically in new Figures S1A and S1C.

We note that MuSC attrition is negligible at 7 days post-TMX, a time when recombination is presumably largely or fully complete. Catenin perdurance must therefore occur to some significant extent. MuSC attrition occurs over the next 3 weeks, and the most likely explanation is that as catenin levels drop below a certain threshold via protein turnover, cells enter the activation and differentiation pathway we document in the paper. Cells which stably remain at 28 days post-TMX may persist either because they: 1) sustained incomplete recombination (maintenance of even 1 of 4 alleles may perhaps be sufficient); or 2) represent a subset of MuSCs in which complete recombination has occurred but the catenin proteins perdure for a very long time.

© 2024. Published by The Company of Biologists under the terms of the Creative Commons Attribution License (https://creativecommons.org/licenses/by/4.0/).

To distinguish between these possibilities, we performed the suggested PCR experiments. qPCR analyses were performed for the Ctnna1 and Ctnna3 loci on MuSCs isolated from the hindlimbs of control and a-cdKO mice 7 days post-TMX, a time when cell attrition has not yet occurred but recombination is presumably largely or fully complete. We used our genotyping primers with qPCR to attempt to quantify loss of the floxed allele band in TMX-treated mice vs. non-treated control mice (we note that appearance of a band representing the Cre-recombined locus is not useful for determining the percentage of recombination that has occurred). We found that, although one major band is seen in saturation PCR reactions run on gels, the qPCR profile revealed shoulders and lower levels of additional non-specific products. Therefore, we did not find this approach accurately quantitative. We also attempted to do this by quantifying bands on gels against other, control, PCR-amplified products but this was, again, not convincingly quantitative. It should be mentioned that what is being attempted is difficult by its nature. While our quantification of MuSC numbers and catenin production by IF is at the single cell level, the PCR approach requires analyzing many pooled cells that carry 0, 1, or 2 copies of the floxed (non-recombined) locus to generate an average value. Combined with the PCR issues described above, this approach has not provided useful information.

In conclusion, our data do not allow us to fully distinguish between the two possibilities listed above (although the former seems most likely). The new results in Figures S1A and S1C are, however, consistent with our previous conclusion that recombination was relatively efficient, despite the perdurance of protein and maintenance of MuSC numbers in earlier time points after recombination. We have modified the text to further explain our interpretation (lines 145-150 and 170-173). We hope that the reviewer will agree that further work of this type will not shed additional light on the issue, and that our conclusions remain justified.

2. The authors present data that a-cdKO and bg-cdKO mice display incomplete regeneration and do not efficiently reoccupy the niche, but the conclusions should be supported by additional data.

a. It is not clear whether the observation that skeletal muscle regeneration is delayed is in consequence to depletion of the stem cell pool or due to MuSCs that are defective for catenins. This is potentially addressed with similar acute ctx injury experiments, but at timepoints before the MuSC pool is depleted, eg/ day 7.

We again thank the reviewer for their suggestion but, based on the new data provided in response to point 1, this is a nearly impossible question to address. Although no MuSCs have been lost at day 7 post-TMX, new Figures S1A and S1C demonstrate that all MuSCs also still express both targeted catenin proteins. Furthermore, quantification of the levels per cell of the various catenin proteins is not possible in a truly rigorous manner. It seems very likely that the incomplete regeneration phenotype is due to a combination of MuSC depletion and remaining, partially catenin-deficient MuSCs that are less able to contribute to regeneration as compared to wild type MuSCs. We have now stated this more directly in the revised manuscript (lines 234-239).

b. The conclusions would be supported by reporting on the identity of MuSCs reoccupying the niche as wt or mutant. For example, if a greater proportion of MuSCs that are present 28d after CTX injury still express the floxed allele, stronger conclusions can be made. This concern is potentially addressed using available antibodies.

This is a great experiment that we had hoped to succeed with. Ideally this would be assessed in vivo but, unfortunately, none of the catenin antibodies we used successfully in single myofiber preparations worked for us on muscle sections. The single myofiber preparation is also problematic in that MuSCs that are not under the basal lamina (as found in mutant animals 28 days post-injury) are largely lost during isolation of myofibers. Therefore these preps lack a critical control population of MuSCs for comparison with those that reoccupy the niche.

Taking our responses to comments 1 and 2 together, we respectfully submit that attempting to go farther in parsing MuSC loss via incomplete recombination vs. catenin perdurance could lead to error-prone work, without changing the major conclusions of the paper. We hope the reviewer will agree.

© 2024. Published by The Company of Biologists under the terms of the Creative Commons Attribution License (https://creativecommons.org/licenses/by/4.0/).

3. In Figure 1, please report the timepoints of the IF analyses.

This is now reported in the legend.

4. Figure 2, Figure 5A,D. For completeness please include scale bars on all images when wt vs mutant muscle is compared. (the reader would otherwise need to assume that the scale between images is the same)

Scale bars are now included in referenced panels.

The following minor concerns and/or suggestions can also be addressed by the authors, but I consider these non-essential revisions.

1. The authors likely did not attempt to support their conclusions with ex vivo analyses of wt vs mutant MuSCs because these analyses would eliminate the important niche interactions, but the authors might consider the following:

a. Ex vivo analysis of MuSCs in culture would remove the MuSCs from their adhesion interactions. With the genetic tools already in hand, the authors could use analyses ex vivo to support their conclusions that the catenins are important for mediating adhesion interactions and not signaling pathways described on pg 6, line 160-161.

b. Ex vivo analysis of wt vs mutant MuSCs present on cultured single EDL myofibres (partial niche with the myofiber intact) would inform on ability of MuSCs to differentiate and/or self-renew, potentially addressed with antibodies against PAX7 and/or myogenin.

We thank the reviewer for understanding why we chose not to pursue these questions ex vivo or in vitro. The differences in response to disruption of cadherin-based adhesion of cells in such preparations vs. in vivo MuSCs is substantial. As one example, MuSCs lacking N- and M-cadherins are able to repair muscle injury normally, including the myoblast fusion step. In contrast, when MuSCs from these same animals are placed in culture, they are fully deficient at myoblast fusion (Goel et al., 2017 in the references). We therefore elected to perform virtually all the functional experiments in this paper using in vivo preparations.

2. Figure 4AB, do MuSCs that lose projections in consequence to a-cdKO also lose the localization or expression of M-cadherin?

Unfortunately, the M-cadherin antibody did not perform well on single myofiber preparations in our hands. However, we did demonstrate in the following panels (Figures 4D and E) that ~75% of a-cdKO MuSCs lose localization and/or expression of M-cadherin in vivo. Because virtually all a-cdKO MuSCs have either no or significantly shorter projections (Figures 4A and B), the concordance of M-cadherin perturbation and projection shortening must be very high.

3. Given the model that MuSCs that do not express catenins escape quiescence, proliferate, differentiate and fuse with the myofiber, it would be interesting to report on potential for myonuclear accretion (myonuclei per myofibre) and/or myofibre size (myofibre CSA) in uninjured mice and/or the contralateral uninjured hindlimb. (If extensive proliferation of activated MuSCs does not occur, one might not expect a biologically significant result and so this interest is not considered an essential revision to the manuscript.)

We noted in the original manuscript that no clusters of BrdU+ MuSCs were visualized during IF analyses, suggesting that MuSC proliferation was very limited (lines 403-408 in the revised manuscript). We have now measured myofiber size in control, a-cdKO, and b/g-cdKO mice. As the reviewer suggests, average minimum myofiber feret diameter at 28 days post-TMX was not different between control and mutant animals. These data are reported as new Figure S1B and S1D.

© 2024. Published by The Company of Biologists under the terms of the Creative Commons Attribution License (https://creativecommons.org/licenses/by/4.0/).

4. Pg 5, line 142-143. Suggestion to report these data graphically.

This suggestion is appreciated and is now incorporated into Figures S1A and S1C.

Reviewer 2 Advance summary and potential significance to field

This work follows up on prior results from the Krauss lab which showed that satellite cell-specific loss of m-cadherin (the adhesion molecule which is most highly expressed and has historically been most strongly associated with satellite cells) did not cause significant changes in cell localization or activity either during quiescence or following activation, while deletion of n-cadherin led to loss of quiescence in the absence of injury. While this phenotype was exacerbated by deletion of both m- and n-cadherin, the affected cells nevertheless remained localized in the sublaminar niche, suggesting that niche adhesion and activation may be separable events. In the current manuscript, Hung et al. elegantly demonstrate that this is indeed the case, and in addition show that when satellite cells lose the ability to form cadherin/catenin-dependent adhesions to the myofiber they will differentiate and fuse with the fiber, thus depleting the quiescent satellite cell pool.

In addition to m- and n-cadherins, at least two other cadherins are present at low levels in satellite cells, thus the group chose instead to delete the intracellular mediators of cadherin signaling, separately deleting two pairs of redundant catenins (b- and g- catenin, and aE- and aT-catenin) from satellite cells. In both experiments they observed a gradual loss of quiescent satellite cells over the 4 weeks following tamoxifen injection- in conjunction with a decrease in sublaminar Pax7+ cells, an accumulation of Pax7+MyoD+ sublaminar cells as well as MyoD+Ki67+ myoblasts in the interstitial space. The remaining satellite cells, when challenged by barium chloride injury, were blunted in their regeneration capacity, although numerically they should have been sufficient. Further, following injury, the number of sublaminar satellite cells generated by self-renewal was equivalent to the diminished population prior to injury rather than the original complement prior to tamixofen. The 'lost' cells were instead located in the interstitial space, leading to the conclusion that cadherin/catenin-dependent adhesion is necessary to reoccupy the niche.

Molecular analysis of satellite cell morphology and membrane adhesions showed that catenin dKO satellite cells lacked or had diminished membrane protrusions, which the Krauss lab has characterized as a property of quiescent satellite cells; expression and/or localization of m-cadherin is also lost in a significant fraction of sublaminar satellite cells in the catenin DKO.

In addition to further defining the role of individual molecules and their interactions in establishment and maintenance of satellite cell quiescence, these studies provide significant new understanding of the redundant and nonredundant roles of cadherins and catenins in critical aspects of satellite cell biology. In particular, they suggest that N-cadherin is specifically required for maintaining quiescence, distinct from the requirement for cadherins in general in maintaining niche adhesion. This may be due to its localization at and activity in the long cellular extensions this lab and others have shown to be associated with maintaining quiescence and mediating activation. The paper is straightforward and well written, and I particularly appreciate the thoughtful treatment of additional aspects of satellite cell biology which loss of catenins in satellite cells could potentially influence and the discussion of why these are unlikely to directly affect the observed phenotype.

We thank the reviewer for their supportive comments.

Reviewer 2 Comments for the author

While it is not absolutely necessary, if it is feasible it would be nice for the authors to follow up on their suggestion that the sublaminar, unactivated satellite cells which are retained 4 weeks after tamoxifen are examples of incomplete/insufficient deletion of the targeted catenin pairs.

As described in the response to Reviewer 1's comment 1, we have tried to provide rigorous proof for this and have been only partially successful. Ideally we would have assessed this in vivo.

© 2024. Published by The Company of Biologists under the terms of the Creative Commons Attribution License (https://creativecommons.org/licenses/by/4.0/).

Unfortunately, the catenin antibodies used successfully in single myofiber preparations did not work for us on muscle sections. We reprint here our response to reviewer 1's similar comment.

First, we performed IF analysis on a-cdKO and b/g-cdKO myofibers at days 7, 10, and 14 post-tamoxifen (TMX). These results confirm the data we reported in the original manuscript in analyses at day 28: at all time points post-TMX, a majority of remaining MuSCs are positive for one or the other catenin protein in both lines. Stated another way, MuSCs that lose both catenin proteins in either a-cdKO and b/g-cdKO mice do not accumulate substantially, even at earlier time points. All these data are presented graphically in new Figures S1A and S1C.

We note that MuSC attrition is negligible at 7 days post-TMX, a time when recombination is presumably largely or fully complete. Catenin perdurance must therefore occur to some significant extent. MuSC attrition occurs over the next 3 weeks, and the most likely explanation is that as catenin levels drop below a certain threshold via protein turnover, cells enter the activation and differentiation pathway we document in the paper. Cells which stably remain at 28 days post-TMX may persist either because they: 1) sustained incomplete recombination (maintenance of even 1 of 4 alleles may perhaps be sufficient); or 2) represent a subset of MuSCs in which recombination has occurred but the catenin proteins perdure for a very long time.

To distinguish between these possibilities, we performed the suggested PCR experiments. qPCR analyses were performed for the Ctnna1 and Ctnna3 loci on MuSCs isolated from the hindlimbs of control and a-cdKO mice 7 days post-TMX, a time when cell attrition has not yet occurred but recombination is presumably largely or fully complete. We used our genotyping primers with qPCR to attempt to quantify loss of the floxed allele band in TMX-treated mice vs. non-treated control mice (we note that production of a band representing the Cre-recombined locus is not useful for determining the percentage of recombination that has occurred). We found that, although one major band is seen in saturation PCR reactions run on gels, the qPCR profile revealed shoulders and lower levels of additional non-specific products. Therefore, we did not find this approach accurately quantitative. We also attempted to do this by quantifying bands on gels against control PCR-amplified products but this was, again, not convincingly quantitative. It should be mentioned that what is being attempted is difficult buy its nature. While our quantification of MuSC numbers and catenin production by IF is at the single cell level, the PCR approach requires analyzing many cells that carry 0, 1, or 2 copies of the floxed (non-recombined) locus to generate an average value. Combined with the PCR issues described above, this approach has not provided useful information.

In conclusion, our data do not allow us to fully distinguish between the two possibilities listed above (although the former seems most likely). The new results in Figures S1A and S1C are, however, consistent with our previous conclusion that recombination was relatively efficient, despite the perdurance of protein and maintenance of MuSC numbers in earlier time points after recombination. We have modified the text to further explain our interpretation (lines 145-150 and 170-173). We hope that the reviewer will agree that further work of this type will not shed additional light on the issue, and that our conclusions remain justified.

Reviewer 3 Advance summary and potential significance to field

In this study, Hung and colleagues generated murine models of muscle stem cell specific, conditional knockouts, of catenin encoding genes to study cadherin-deficiency in homeostasis and regeneration. Using a combination of in vivo, ex vivo and single cell RNA-sequencing approaches, the authors describe a cadherin-dependant mechanism for satellite cell adhesion and maintenance of quiescence within the niche. This study is original and sheds light on important mechanisms for satellite cell niche localisation and regenerative capacity. It also represents a considerable body of work, not least because of the use of multiple knockout lines of mice. Additionally, the authors carried out a characterisation of the fate of cadherin-deficient MuSC and revealed that satellite cell attrition occurs via precocious differentiation, re-entry to the niche and fusion to myofibres. Altogether, this article brings interesting observations that will benefit the muscle and stem cell communities.

We thank the reviewer for their supportive comments.

© 2024. Published by The Company of Biologists under the terms of the Creative Commons Attribution License (https://creativecommons.org/licenses/by/4.0/).

Reviewer 3 Comments for the author

The flow of the study is very logical and does not suffer from any major technical issue, only a few minor comments are listed below:

In Fig.1 and 2, the authors described a decline in Pax7+ MuSC numbers in alpha-cdKO and betagamma-cdKO mice. They confirmed that satellite cell attrition was not the consequence of apoptosis using TUNELassay and cleaved-caspase 3 staining in Fig.S5. However, could a proportion of these cells be senescent and therefore, not contributing to muscle regeneration? That could explain the regeneration deficit and CSA decrease in catenin-deficient mice.

We thank the reviewer for bringing up a helpful point we did not consider in the initial manuscript. To address this experimentally, sections from control and a-cdKO mice at 14 days post-TMX were stained for SA-bGal using the SPiDER-bGal reagent. Muscles from wild type mice 7dpi were used as a positive control. No cells were positive for SPiDER-bGal in either control or a-cdKO sections, whereas robust fluorescence was detected in the control 7dpi muscles. We conclude that senescence is not a significant mechanism of MuSC attrition in a-cdKO muscles. These data have been added as Figure S6C and are described in lines 360-364.

In Fig.3, the authors performed single cell RNA-sequencing of lineage traced Pax7Cre-tdT mice in controls and alpha-cdKO mice. Have the authors considered performing histology analysis of the skeletal muscles of these mice at 14 or 28 days post-tamoxifen? As a majority of quiescent satellite cells are lost due to precocious differentiation and fusion to existing myofibres, this could also be confirmed by in an increased number of Tomato+ fibres in alpha-cdKO mice. Some simple histology analyses in the knockout model would provide strongly confirmatory data for the authors model of the fate of satellite cells released from quiescence.

We thank the reviewer for this suggestion and agree that, in principle, this type of experiment could provide useful information. However, we were aware that others have published that even in control, uninjured mice, MuSCs contribute to adult muscle fibers, and that this occurs mostly in the absence of nucleotide analog incorporation (Pawlikowski et al., Skeletal Muscle 5:42, 2015; Keefe et al. Nat Commun 6:7087, 2015). We observed the same phenomenon in attempting the reviewer's suggested experiment. Pax7tdTom (control) and a-cdKO mice carrying the R26tdTom allele were injected with TMX as described in the Methods, and TA muscles were harvested 14 days post-TMX. Unfortunately, the pervasive labeling of control myofibers with tdTomato interfered with our ability to observe a clear difference from the α-cdKO fibers. As reported in the original manuscript, BrdU+ MuSCs were very rare in control mice, but easily detected in a-cdKO mice. This suggests that there are mechanistic differences between how MuSCs contribute to fibers in control vs. a-cdKO mice, but hope the reviewer will agree that analysis of this phenomenon is beyond the scope of the current manuscript.

In Fig.3D, the table show the percentage of cells belonging to the control or alpha-cdKO sample, present in each of the defined clusters, based on the Harmony integrated UMAP. The authors are claiming a significant increase in the number of cycling and differentiated cells in the alpha-cdKO sample, however, in Fig.S4A, the number of cells in alpha-cdKO samples (alpha--cdKO-1, alpha--cdKO-2) is almost twice as the ones in control samples (Control-1, Control-2). Are the authors normalising to the total number of cells?

We apologize for the confusion on this point and have edited the legend to Fig. 3 for clarity. The percentage contribution in each cluster is indeed calculated by normalizing to the genotype contribution to the total number of cells analyzed.

While it is expected and supported by the authors immuno-histochemistry data that the number of differentiated cells are increased in alpha-cdKO samples, it is surprising that the number of alpha-cdKO cells are decreased as compared to the controls in the cluster 5, corresponding to mature myocytes. Have the authors considered performing differential expression analysis between control and alpha-cdKO groups to characterise a potential shift in cell identity or gene compensation mechanisms?

© 2024. Published by The Company of Biologists under the terms of the Creative Commons Attribution License (https://creativecommons.org/licenses/by/4.0/).

Differential expression analysis was performed between control vs. a-cdKO groups in cluster 5. Only three genes were upregulated in a-cdKO samples: 1) tropomyosin 3 related sequence 7 (tpm3-rs7); 2) predicted pseudogene 8797 that resembles a ubiquitin B sequence (gm8797); and 3) Xist, due to the a-cdKO samples coming from female mice. We speculate that when the samples were isolated 14 days post-TMX, a proportion of a-cdKO cells that would have contributed to cluster 5 had already fused with their adjacent myofiber, reducing the number of cells that could contribute to this cluster in comparison to co

---

Second decision letter

MS ID#: DEVELOP/2023/202387

MS TITLE: Cadherin-dependent adhesion is required for muscle stem cell niche anchorage and maintenance

AUTHORS: Margaret Hung, Hsiao-Fan Lo, Aviva G. Beckmann, Deniz Demircioglu, Gargi Damle, Dan Hasson, Glenn L. Radice, and Robert S. Krauss
ARTICLE TYPE: Research Article

I am happy to tell you that your manuscript has been accepted for publication in Development, pending our standard ethics checks.

Reviewer 1

*Advance summary and potential significance to field*

This study explores the critical role of adhesion interactions between adult tissue-specific stem cells and their microenvironment, or niche. The research group’s previous work has shown that muscle stem cell (MuSC) adhesion to the myofibre is partly facilitated by cadherin-based interactions. However, the significance of these interactions in maintaining MuSCs in a state of quiescence had not been established, possibly due to the overlapping functions of various cadherins expressed at different levels.

To investigate whether cadherin interactions are key to maintaining MuSC quiescence, the researchers employed genetic tools in mice to eliminate catenins the cytoplasmic proteins that are essential to mediate cadherin based adhesion.

The findings reveal that without catenins, MuSCs become active, leave their niche and eventually get depleted due to premature differentiation and integration into muscle fibers.

In this revised version of the manuscript, the authors have added new immunofluorescence analyses of catenins at 7, 14 and 21 days after tamoxifen administration, which reports both on the efficiency of recombination and the extent to which catenins are depleted over time. In addition, the authors present new additional data showing that senescence is not a significant contributor to the observed phenotypes. Altogether the data support the manuscript's conclusions that catenin deficiency leads to MuSC activation, exit from quiescence, and eventual depletion through premature differentiation.

*Comments for the author*

The authors have made a sincere effort to respond to my comments through additional experimentation. They have reported new data that shows the efficiency of catenin depletion over time after the course of tmx administration. Moreover, I appreciate that the authors are forthcoming about limitations encountered due to technical challenges or experimental constraints during their revisions.

© 2024. Published by The Company of Biologists under the terms of the Creative Commons Attribution License (https://creativecommons.org/licenses/by/4.0/). 

This is a very nice manuscript with high quality data supporting a model by which catenin deficiency leads to MuSC activation, exit from quiescence, and eventual depletion through premature differentiation. I congratulate the authors on a nice study and their novel contribution to the field.

Reviewer 2

*Advance summary and potential significance to field*

In addition to further defining the role of individual molecules and their interactions in establishment and maintenance of satellite cell quiescence, these studies provide significant new understanding of the redundant and nonredundant roles of cadherins and catenins in critical aspects of satellite cell biology. In particular, they suggest that N-cadherin is specifically required for maintaining quiescence, distinct from the requirement for cadherins in general in maintaining niche adhesion. This may be due to its localization at and activity in the long cellular extensions this lab and others have shown to be associated with maintaining quiescence and mediating activation. The paper is straightforward and well written, and I particularly appreciate the thoughtful treatment of additional aspects of satellite cell biology which loss of catenins in satellite cells could potentially influence and the discussion of why these are unlikely to directly affect the observed phenotype.

*Comments for the author*

The efforts made by the authors to address the question raised by me and another reviewer are sufficient even if they were not entirely successful, and the clarifications in the text describe the rationale and results of those experiments. I would approve this manuscript for acceptance in its current form.

Reviewer 3

*Advance summary and potential significance to field*

In this study, Hung and colleagues generated murine models of muscle stem cell specific, conditional knockouts, of catenin encoding genes to study cadherin-deficiency in homeostasis and regeneration. Using a combination of in vivo, ex vivo and single cell RNA-sequencing approaches, the authors describe a cadherin-dependant mechanism for satellite cell adhesion and maintenance of quiescence within the niche. This study is original and sheds light on important mechanisms for satellite cell niche localisation and regenerative capacity. It also represents a considerable body of work, not least because of the use of multiple knockout lines of mice. Additionally, the authors carried out a characterisation of the fate of cadherin-deficient MuSC and revealed that satellite cell attrition occurs via precocious differentiation, re-entry to the niche and fusion to myofibres. Altogether, this article brings interesting observations that will benefit the muscle and stem cell communities.

*Comments for the author*

The authors have expertly attended to our requested revisions, and attempted to answer all our comments.

Where this has been technically challenging to achieve they have presented rational arguments as to why.

The manuscript is much improved and will make an excellent addition to the published literature.

© 2024. Published by The Company of Biologists under the terms of the Creative Commons Attribution License (https://creativecommons.org/licenses/by/4.0/).
